# The mechanism of activation of the actin binding protein EHBP1 by Rab8 family members

**DOI:** 10.1038/s41467-020-17792-3

**Published:** 2020-08-21

**Authors:** Amrita Rai, Nathalie Bleimling, Ingrid R. Vetter, Roger S. Goody

**Affiliations:** 1 Department of Structural Biochemistry, Max Planck Institute of Molecular Physiology, Otto-Hahn-Strasse 11, 44227 Dortmund, Germany; 2 Department of Mechanistic Cell Biology, Max Planck Institute of Molecular Physiology, Otto-Hahn-Strasse 11, 44227 Dortmund, Germany

**Keywords:** Biochemistry, Biophysics, Cell biology, Structural biology

## Abstract

EHBP1 is an adaptor protein that regulates vesicular trafficking by recruiting Rab8 family members and Eps15-homology domain-containing proteins 1/2 (EHD1/2). It also links endosomes to the actin cytoskeleton. However, the underlying molecular mechanism of activation of EHBP1 actin-binding activity is unclear. Here, we show that both termini of EHBP1 have membrane targeting potential. EHBP1 associates with PI(3)P, PI(5)P, and phosphatidylserine via its N-terminal C2 domain. We show that in the absence of Rab8 family members, the C-terminal bivalent Mical/EHBP Rab binding (bMERB) domain forms an intramolecular complex with its central calponin homology (CH) domain and auto-inhibits actin binding. Rab8 binding to the bMERB domain relieves this inhibition. We have analyzed the CH:bMERB auto-inhibited complex and the active bMERB:Rab8 complex biochemically and structurally. Together with structure-based mutational studies, this explains how binding of Rab8 frees the CH domain and allows it to interact with the actin cytoskeleton, leading to membrane tubulation.

## Abstract (web summary)

EHBP1 is an adaptor protein that regulates vesicular trafficking and links endosomes to the actin cytoskeleton. Here, authors show that both termini of EHBP1 have membrane targeting potential and that in the absence of its binding partner Rab8, the bMERB and CH domain of EHBP1 form an intramolecular complex which auto-inhibits actin binding.

## Introduction

EHBP1 was originally identified as an Eps15-homology domain-containing protein 1/2 (EHD1/2) interacting partner that plays a central role in GLUT4 transport and couples endocytic vesicles to the actin cytoskeleton^1,2^. EHBP1 co-localizes with the actin cytoskeleton and overexpression of either EHBP1 or EHD2 leads to extensive actin reorganization^2^. Disruption of EHBP1/EHDs by siRNA leads to inhibition of transferrin endocytosis and GLUT4 transportation^2^. The Ras superfamily GTPase Rab10 has also been shown to regulate the translocation of GLUT4 in adipocytes^3^. In our previous work, we showed that EHBP1 is an effector molecule for Rab8 family members, including Rab10, and forms complexes with 1:1 stoichiometry^4,5^. Recent work has also shown that a Rab10-EHBP1-EHD2 trimeric complex plays a crucial role in lipid droplet engulfment during lipophagy in hepatocytes^6^. Moreover, *C. elegans* EHBP1 promotes endosomal tubulation by linking the membrane lipid PI(4,5)P2 to the actin cytoskeleton and this interaction is enhanced upon Rab10 binding^7^.

Apart from having roles in vesicular trafficking and autophagy, EHBP1 is implicated in early development and cancer. In *C. elegans*, EHBP1 depletion leads to an endocytic recycling defect in the intestine and in nonpolarized germline cells and the phenotype was recapitulated upon Rab8/10 deletion^8^. *Drosophila EHBP1* has been shown to play an essential role in eye development by regulating the exocytosis of Scabrous, a positive regulator of Notch signaling^9^. Notch signaling has been implicated in metastatic prostate cancer, and a genome-wide SNP association study shows that EHBP1 is associated with aggressive disease^10–13^. EHBP1 controls the invasiveness of PTEN-positive prostate cancer cells and is essential for the anti-invasive effect of the drug atorvastatin^14^.

Despite having information on EHBP1 at the functional level, convincing biochemical data on EHBP1 activation are missing. In this work, we have identified and characterized an auto-inhibited state of human EHBP1, which is mediated by an intramolecular association between the CH domain and the bMERB domain. We have elucidated the structure of the CH domain in complex with the bMERB domain, providing an explanation for the specificity of EHBP1_bMERB_ toward its CH domain. Transient kinetic experiments show that the binding of Rab8 family members to the bMERB domain releases the CH domain. Furthermore, we have solved the crystal structure of the bMERB domain in complex with human Rab8a. Structural analyses supported by mutagenesis and biochemical experiments identify key residues for the interaction and explain why the generation of a stable CH-bMERB-Rab8 ternary complex is not possible. Our biochemical and structural data suggest that the interaction between the C-terminal bMERB domain and the central CH domain has a regulatory role in the function of EHBP1 and binding of Rab8 family members to the bMERB domain releases the CH domain, which can then interact with the actin cytoskeleton.

## Results

### Domain architecture and localization of EHBP1

**Fig. 1:**
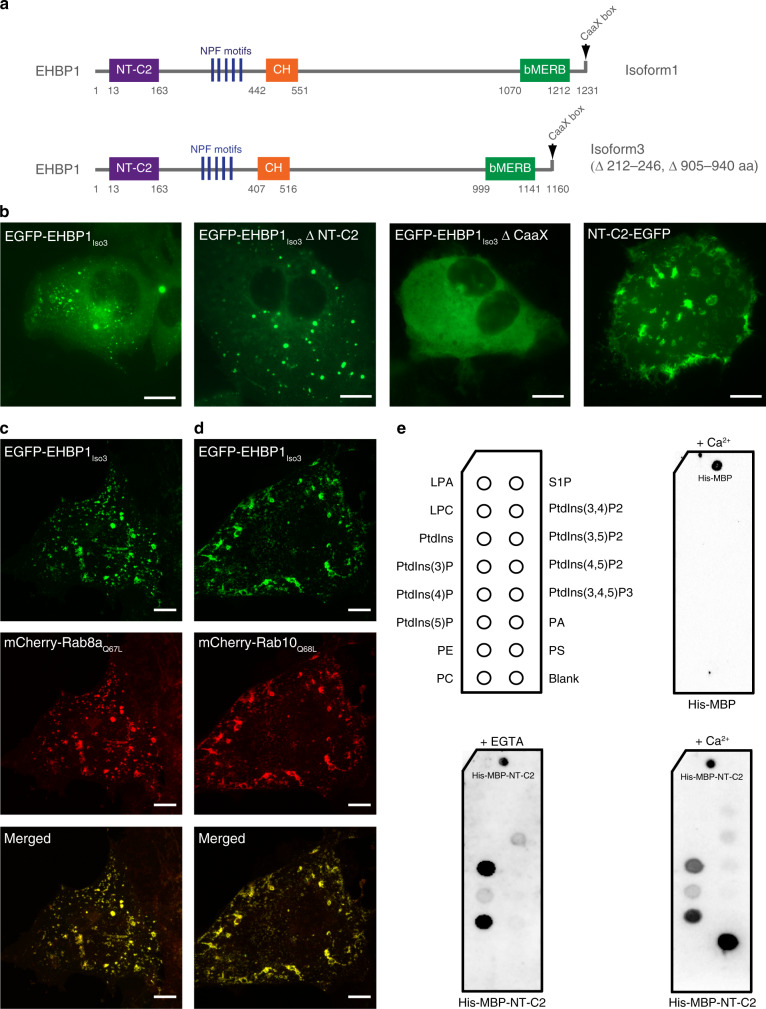
Domain architecture and cellular localization of human EHBP1. **a** EHBP1 contains an N-terminal C2-like domain (NT-C2), a central CH (calponin homology) domain, and a C-terminal a coiled-coil bMERB (bivalent Mical/EHBP Rab binding) domain. At the end of the bMERB domain, EHBP1 also has a C-terminal prenylation motif (CaaX box). In addition to these domains, EHBP1 harbors five NPF motifs. Isoform 1 is composed of 1231 amino acids and isoform 3 lacks residues 212–246 and residues 905–940. **b** Cellular localization of different EGFP tagged EHBP1 constructs. Scale bar: 10 µm. **c**, **d** EGFP-EHBP1 shows strong co-localization with mCherry tagged Rab8a_Q67L_ and mCherry tagged Rab10_Q68L_. Scale bar: 10 µm. Experiments were repeated at least three times independently with similar results. **e** Lipid-binding activity of NT-C2 domain of EHBP1. Lipid spots present on the PIP strip are indicated in the left panel. LPA Lysophosphatidic Acid, LPC lysophosphatidylcholine, PtdIns phosphatidylinositol, PI(3)P phosphatidylinositol-3-phosphate, PI(4)P phosphatidylinositol-4-phosphate, PI(5)P phosphatidylinositol-5-phosphate, PE phosphatidylethanolamine, PC phosphatidylcholine, S1P sphingosine-1-phosphate, PI(3,4)P2 phosphatidylinositol-3,4-bisphosphate, PI(3,5)P2 phosphatidylinositol-3,5-bisphosphate, PI(4,5)P2 phosphatidylinositol-4,5-bisphosphate, PI(3,4,5)P3 phosphatidylinositol-3,4,5-trisphosphate, PA phosphatidic acid, PS phosphatidylserine. Experiments were repeated at least three times independently with similar results.

EHBP1 consists of an N-terminal C2 domain, a central CH, a C-terminal bMERB domain, and a CaaX box at the C-terminus that is a substrate for FTase^4^. The UniPort database has reported three EHBP1 isoforms and for biochemical studies, the domain boundaries are taken from isoform 1 (Q8NDI1-1), whereas for cellular localization experiments, fluorescent constructs are based on isoform 3 (Q8NDI1-3) (Fig. 1a). Previously, we have reported that the bMERB domain, together with the CaaX box, is sufficient to target an EGFP-fusion protein to the endosome and co-localize with active Rab8/10^4^. Here, we show that the full-length protein, as well as the construct lacking the NT-C2 domain, is targeted to structures that appear to be endosomes. Surprisingly, the EGFP-EHBP1_ΔCaaX_ construct is cytosolic, suggesting that even in the full-length background, the CaaX box is indispensable for endosomal localization. However, this could be due to the presence of the N-terminal EGFP tag, which could hinder membrane association via the NT-C2 domain. To rule out this possibility, we expressed an isolated NT-C2 EGFP (C-terminal) fusion construct and showed that this construct is cytosolic as well as having the potential to associate with membranous structures (Fig. 1b). Thus, our localization data suggest that both termini of EHBP1 have membrane targeting potential. Further, we could show that the full-length EHBP1 co-localizes with Rab8/10 active constructs (Fig. 1c, d).

Intrigued by the observation that the NT-C2 domain associates with membranes, we investigated the lipid-binding properties of the domain by performing a protein-lipid overlay assay using the His6-MBP-NT-C2 domain fusion protein. The NT-C2 domain of EHBP1 is a member of a unique but not well studied NT-C2 domain family^15^, and in general, most of the C2 domain family members are known to interact with phospholipids directly^7,15,16^. Since most of the C2 domain family members have been reported to be regulated by Ca^2+^ ions, we performed the experiments in the presence or absence of Ca^2+^ ions. The results obtained indicated that the NT-C2 domain of EHBP1 binds to phosphatidylserine in the presence of Ca^2+^ but also binds PI(3)P and PI(5)P in a Ca^2+^ independent manner (Fig. 1e). Additional biochemical experiments are required to validate and extend these initial observations.

### C-terminal high affinity Rab-binding site of bMERB domain

**Fig. 2:**
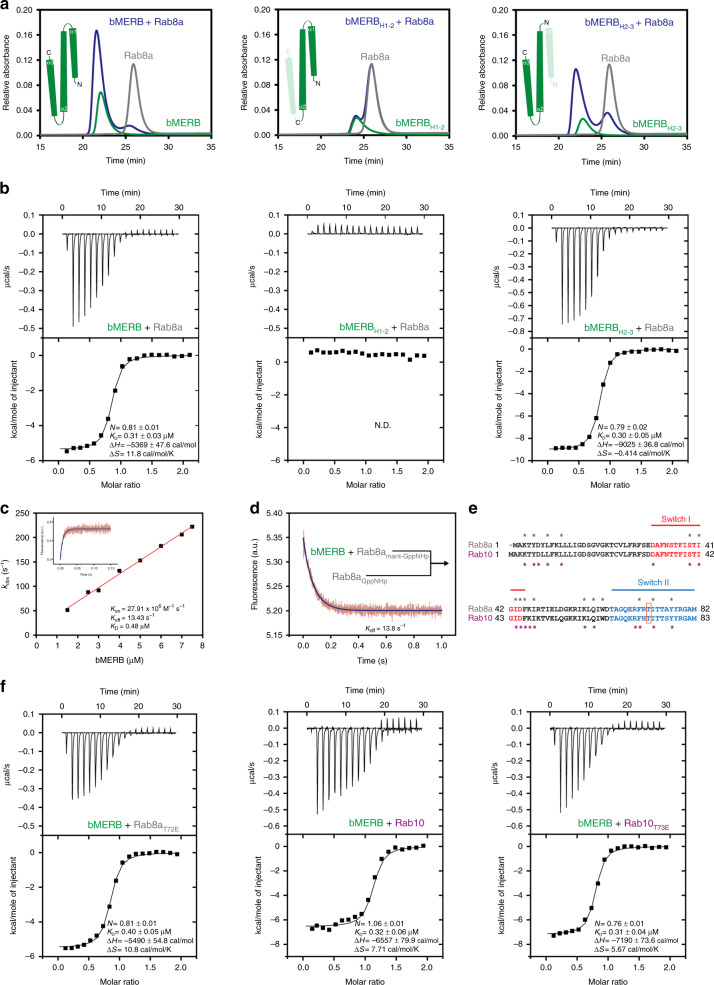
Rab8 preferentially binds to the C-terminal Rab-binding site of the bMERB domain. **a** The bMERB domain (green), GppNHp Rab8a_1-176_ (gray), and a mixture of both (blue) were loaded onto a Superdex 75 10/300 GL column and monitored for complex formation. Complex formation was observed in the case of the full-length domain (indicated as green helices in the inset) as well as a construct lacking the N-terminal helix, indicating that helices 2–3 are crucial for Rab8a_GppNHp_ interaction, clearly showing that only the high-affinity C-terminal Rab8 binding site is present in the EHBP1 bMERB domain. **b** Binding affinities were measured by titrating GppNHp Rab8a_1-176_ (500 µM) to the bMERB domain (50 µM). Integrated heat peaks were fitted to a one-site-binding model yielding the binding stoichiometry (N), the enthalpy (ΔH), the entropy (ΔS), and the dissociation constant (*K*_D_). The data are representative of at least three repetitions. N.D. denotes not detected. **c** Observed pseudo first order association rate constants between 0.5 μM mantGppNHp Rab8_1-176_ and different concentrations of the bMERB domain (1.5–8 µM). Assocation was monitored by the change in fluorescence intensity using a stopped-flow apparatus at 25 °C. Association of the bMERB domain with mantGppNHp Rab8_1-176_ leads to an increase in intensity. As an example of the data obtained, the association between 0.5 μM mantGppNHp Rab8_1-176_ and 4 μM of the bMERB domain is shown in the inset. **d** Dissociation of Rab8a from the bMERB domain was monitored using the decrease of fluorescence after mixing a complex of mantGppNHp Rab8a_1-176_ with the bMERB domain (2 μM) with a 20-fold excess of unlabeled GppNHp Rab8a_1-176_. **e** Sequence alignment of Rab8a/10, using Clustal Omega. Switch I/II regions are indicated in red and light blue colors, respectively. The residues involved in binding with the Mical cL bMERB domain are denoted by gray (Rab8a) and magenta asterisks (Rab10), and T72/73 phosphorylated by LRRK2 is shown in the orange box. **f** Phosphomimetic mutation of switch II threonine does not affect bMERB binding of both Rab8 and Rab10. Binding affinities were measured by ITC experiments. The data are representative of at least three repetitions.

Previously, we reported that the EHBP1 bMERB domain preferentially binds to Rab8 family members^4^. In this work, we have used a smaller version of the bMERB_1060-1212_ domain containing all three predicted α-helices^4^, which, consistent with our prior work, forms a stable complex with Rab8a with a similar affinity to the longer bMERB_1047-1220_ construct. Earlier, we reported that some bMERB family members have two Rab-binding sites, a high-affinity C-terminal binding site, and a lower or similar affinity N-terminal binding site^4,5^. However, in the case of the EHBP1 bMERB domain, we observed only a single-binding site^4^. To localize the exact binding site, we made two deletion constructs in which either the N or the C-terminal helix was deleted (Fig. 2a, insets). Both the full-length as well as the N-terminally truncated constructs form stable complexes with Rab8a as observed by analytical size exclusion chromatography (aSEC) experiments. Further, isothermal titration calorimetry (ITC) measurements show that both bMERB constructs bind to Rab8a:GppNHp with a *K*_D_ value of 0.3 µM (Fig. 2b). In contrast, we could not detect any interaction with the (potential) N-terminal low-affinity binding site.

Next, we independently measured the association (*k*_on_) and dissociation rate (*k*_off_) constants for *K*_D_ calculation (*K*_D_ = *k*_off_/*k*_on_). For *k*_on_ measurements, association kinetics of Rab8a loaded with fluorescent 2′3′-O-(N-methyl-anthraniloyl) mantGppNHp were monitored with increasing concentrations of the bMERB_1060-1212_ domain using a stopped-flow apparatus. *k*_on_ for Rab8a_mantGppNHp_ is 2.79 × 10^7^ M^−1^ s^−1^ and the intercept on the y-axis yielded the *k*_off_ (13.4 s^−1^) (Fig. 2c). Direct *k*_off_ was determined by the displacement of the Rab8a_mantGppNHp_ from the bMERB_1060-1212_ domain by an excess of Rab8a_GppNHp_, *k*_off_ of 13.8 s^−1^ was observed (Fig. 2d) and *K*_D_ value of 0.48 µM was calculated, in reasonable agreement with the value of 0.31 µM obtained in ITC experiments.

### Phosphomimetic mutation of Rab8/10 switch II threonine

LRRK2, a serine/threonine kinase phosphorylates threonine of switch II of Rab8a_T72_/10_T73_. Phosphorylated Rabs are GDI resistant, thus increasing their lifetime on the membrane^17,18^. Further, phosphorylation also increases Rab8a/10 binding to the effector proteins RILPL1/L2^18^. Structure-based sequence alignment shows that the switch II threonine of Rab8a/10 does not directly interact with Mical1_bMERB_ and Mical cL_bMERB_^4^ (Fig. 2e). However, it is still unknown whether phosphorylation of Rab8a/10 has any effect on the EHBP1_bMERB_ interaction. Therefore, we prepared the phosphomimetic mutants Rab8a_T72E_/10_T73E_ by site-directed mutagenesis and checked for complex formation with the bMERB domain by aSEC/ITC experiments (Fig. 2f and Supplementary Fig. 1). No effect of phosphomimetic mutations of Rab8a/10 on the EHBP1_bMERB_ interaction was observed.

### CH and bMERB domain association and release by Rab8

**Fig. 3:**
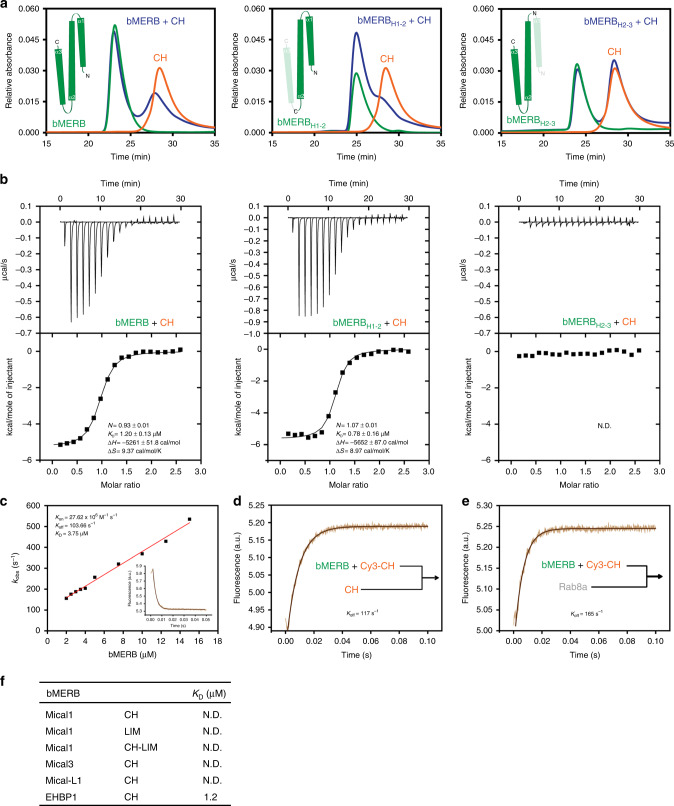
Interaction between the CH and the bMERB domain of EHBP1 and disruption by Rab8. **a** The bMERB domain (green), CH domain (orange), and a mixture of both (blue) were loaded onto a Superdex 75 10/300 GL column to monitor for complex formation. Clear complex formation was observed for the construct lacking the C-terminal helix (indicated as green helices in the insets). However, in the case of the full-length construct, only a shift in the CH domain peak was observed, indicating distribution between free and EHBP1 bMERB bound CH domain at the concentrations used. No complex formation or shift in the CH peak was observed for the construct lacking the N-terminal helix. **b** Binding affinities were measured by titrating the CH domain (800 µM) to either the full length or N/C-terminally truncated bMERB domain (60 µM). Integrated heat peaks were fitted to a one-site-binding model yielding the binding stoichiometry (N), the enthalpy (ΔH), the entropy (ΔS), and the dissociation constant (*K*_D_). The data are representative of at least three repetitions. **c** Observed association first-order rate constants between 0.5 μM Cy3 labeled CH domain with different concentrations of full-length bMERB domain (2–16 µM). Kinetics were registered as a change in fluorescence using a stopped-flow apparatus at 25 °C. Association of bMERB to the Cy3-CH leads to a decrease in the fluorescence. As an example, the kinetics of association between 0.5 μM Cy3 labeled CH domain and 5 μM of bMERB domain is shown in the inset. **d**, **e** Dissociation of the CH domain was measured by monitoring the increase of fluorescence after mixing a complex of Cy3 labeled CH with full-length bMERB domain (2 μM) with a 20-fold excess of either unlabeled CH domain or GppNHp Rab8a. **f** Results of systematic analysis of interactions between the bMERB domains of different family members with their respective CH/LIM/CH-LIM domains (from Supplementary Fig. 2). Interactions were measured by ITC. N.D. denotes not detected.

It has been suggested that some bMERB family members exist in an auto-inhibited state in the absence of Rab and that the bMERB domains have to be released or exposed for activation to occur^19–24^. Since it was was difficult to investigate a possible intramolecular interaction between the CH and bMERB domains of EHBP1 using constructs containing both domains, we examined the interaction between the separately purified bMERB and CH domains of EHBP1, initially employing aSEC experiments. Clear complex formation with the CH domain was observed for the construct lacking the C-terminal helix of the bMERB domain (Fig. 3a). However, in the case of the full-length construct, only a partial shift in the CH domain peak was observed, indicating a weaker equilibrium between free and bMERB-bound CH domains. No complex formation was observed for the construct lacking the N-terminal helix (Fig. 3a). Using ITC measurements, binding was observed between full-length bMERB domain and CH domains and similar to Rab8a, CH domain binding to bMERB domain is an enthalpy driven process with a *K*_D_ value of 1.2 µM and stoichiometry of 1:1. The C-terminally truncated construct showed a slightly higher affinity (*K*_D_ = 0.78 µM), whereas no binding was observed for the construct lacking the N-terminal helix, clearly indicating that helices 1 and 2 constitute the CH-binding site (Fig. 3b).

Using transient kinetic measurements, we determined the *k*_on_ and *k*_off_ rate constants for the CH and full-length bMERB domain interaction. The observed pseudo first oder association rates of Cy3-CH were plotted against increasing concentrations of the bMERB domain and a *k*_on_ of 2.76 × 10^7^ M^−1^ s^−1^_,_ while *k*_off_ was 103.7 s^−1^ as obtained from the y-axis intercept (Fig. 3c). *k*_off_ was also measured directly by displacing Cy3-CH from the bMERB domain by mixing with an excess of unlabeled CH domain, leading to a value of 117 s^−1^ (Fig. 3d). These values led to a calculated *K*_D_ value of 3.75–4.23 µM. This is higher than the value obtained by ITC, suggesting some interference with bMERB binding by the Cy3 label. We note that the association rate constant for CH and Rab8a binding to bMERB is nearly identical, whereas the *k*_off_ for CH is ca. ten times higher than for Rab8a (Fig. 2c, d).

To test whether binding of Rab8a to the bMERB domain can release the CH domain, we generated the Cy3-CH:bMERB complex and mixed it rapidly with an excess of Rab8a. This led to an increase in fluorescence intensity, indicating that Rab8a can indeed displace the CH domain and *k*_off_ was 162 s^−1^ (Fig. 3e), which is significantly larger than that for spontaneous dissociation (Fig. 3d), suggesting an active displacement mechanism via a ternary complex between the three proteins.

In similar experiments using different bMERB domains and their respective CH, LIM, or CH-LIM domains, no complex formation was detected using aSEC/ITC experiments (Fig. 3f and Supplementary Fig. 2).

### The CH domain interacts with actin filaments

**Fig. 4:**
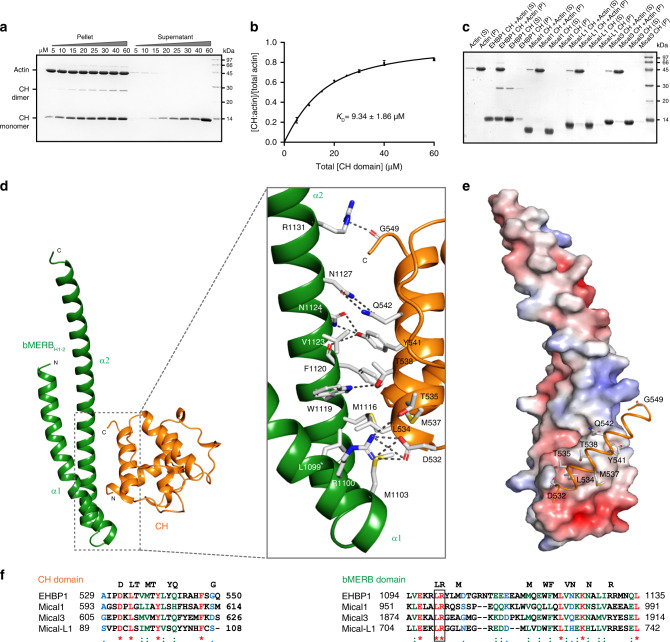
CH domain interaction with F-actin and structure of the EHBP1 CH:bMERB_H1-2_ complex. **a** The EHBP1 CH domain co-sediments with F-actin filaments in vitro. SDS-PAGE of pellets and supernatants from high-speed centrifugation performed at a fixed concentration of F-actin (10 µM) and with varying concentrations of the EHBP1 CH domain (0–60 µM) is shown. **b** The normalized fraction of F-actin bound EHBP1 CH domain as a function of total EHBP1 CH domain concentration. Values were calculated from densitometry of SDS-PAGE. The data from two technical repeats are shown as means ± s.d. (*n* = 2). The error bars are included in the plot but are too small to be displayed on several of the points. **c** Results of systematic analysis of interactions between the CH domains of different bMERB family members with F-actin via co-sedimentation experiments. Only the EHBP1 CH domain interacts with F-actin. Experiments were repeated at least two times independently with similar results. Source data are provided as a Source Data file. **d** Cartoon representation of the CH:bMERB_H1-2_ complex structure. The bMERB domain is colored green and the CH domain in orange. The inset shows the zoom-in overview of the CH:bMERB interaction interface. Hydrogen bonds and polar interactions are shown in gray dashed lines. **e** Surface electrostatic potential of bMERB_H1-2_ calculated in PyMOL using the APBS-PDB2PQR plugin and visualized in red to blue (−5 kT/e to +5 kT/e). The C-terminal helix of the CH domain is shown. **f** Sequence alignment of the interacting regions of the CH and the bMERB domain of different bMERB family members. Residues directly involved in the CH:bMERB interactions are shown over the top of sequence alignment and the conserved LR motif is shown in the black box.

To serve as an actin-binding domain (ABD), a tandem repeat of CH1 and CH2 is usually required^25^. The CH1 domain directly interacts with F-actin, while CH2 plays a supporting role^26^. The human EHBP1 CH domain is quite similar to the CH2 domain of alpha-actinin4 (36% identity), which usually has a lower actin-binding affinity; however, the *C. elegans* CH^EHBP1^ domain was shown to interact with actin filaments^7^. Using actin co-sedimentation assays, we could demonstrate an interaction with the human EHBP1 CH domain, with a *K*_D_ value of 9.34 ± 1.86 µM (Fig. 4a, b). The affinity is relatively high for a single CH2 domain; for alpha-actinin and utrophin isolated CH2 domains a *K*_D_ of >1000 µM was reported^27,28^.

A similar interaction of the CH domain from Micals/Mical-like family members could not be detected (Fig. 4c).

### The overall structure of the CH:bMERB complex

Although crystals of the EHBP1 CH:bMERB diffracting to 4.0 Å were obtained, these were twinned and the crystal quality could not be improved. Since we had noted earlier that helices 1 and 2 of the bMERB domain are sufficient to form a complex with the CH domain, we then used the bMERB_H1-2_:CH complex and obtained crystals (SG: P 2_1_) diffracting to 2.2 Å. The structure was solved as described in material and methods (Data and refinement statistics are shown in Supplementary Table 1).

The asymmetric unit contained two copies of the CH:bMERB_H1-2_ complex, sharing the same overall architecture (Supplementary Fig. 3a, b). According to a DALI search^29^ against the protein data bank, the EHBP1 CH domain is most similar to the CH2 domains of alpha-actinin4^30^ and beta-spectrin^31^ (Supplementary Fig. 3c). As expected, the bMERB_H1-2_ is composed of two helices and the CH domain adopts a similar fold to the corresponding free EHBP1 CH domain (PDB 2D89)^32^ (Supplementary Fig. 3c). The interface of the bMERB_H1-2_:CH complex shows both hydrophobic and hydrophilic interactions with a buried surface area of 593 Å^2^ (Fig. 4d, e). Most of the interactions with the CH domain lie on α-helix 2, with some additional interacting residues provided by α-helix 1 (Fig. 4d). An array of hydrophobic residues including L1099, M1103 of α-helix 1 and M1116, W1119, F1120 and V1123 from α-helix 2 forms a contiguous hydrophobic patch on the bMERB surface with extensive contacts to L534, M537, and Y541 of the C-terminal helix of the CH domain (Fig. 4d, e). Besides these hydrophobic interactions, several polar interactions were observed at the CH:bMERB binding interface, including D532^CH^-R1100, T535^CH^-R1100, T538^CH^-W1119, Y541^CH^-N1124, and Q542^CH^-N1127. The Y541^CH^ side chain also forms a hydrogen bond with the backbone carbonyl of F1120, and the R1131 side chain forms another hydrogen bond with the carbonyl of G549^CH^ (Fig. 4d inset). Some of the interface residues are conserved in different bMERB family members (Fig. 4f).

### Key elements of the CH:bMERB interface

**Fig. 5:**
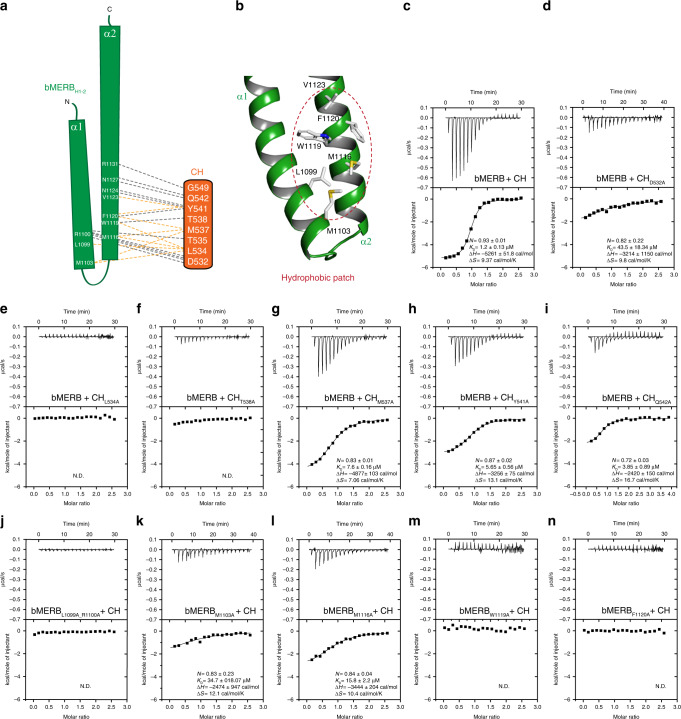
The N-terminal hydrophobic patch of bMERB domain is essential for the CH domain interaction. **a** Schematic illustration of the interactions between the bMERB_H1-2_ domain and the CH domain C-terminal helix. Hydrophobic interactions are indicated by light orange dashed lines, ionic interactions, and H-bonds are indicated by gray dashed lines. **b** Hydrophobic residues at the CH-binding site of the EHBP1 bMERB domain. **c**–**n** Mutational alanine screening of the CH:bMERB complex binding interface via ITC measurements. Mutation of the CH domain residues that are involved in stabilization of the hydrophobic patch results in a significant defect in binding and the integrity of the hydrophobic patch is crucial for the CH interaction. The data are representative of at least three repetitions. N.D. denotes not detected.

To identify the crucial key residues required for complex formation, we purified a series of CH as well as bMERB domain mutants and checked their interaction using ITC measurements. Beginning with the C-terminal interacting residues of the CH domain, we could show that mutation of the conserved D532 to alanine leads to a more than 40-fold reduction in binding affinity (Fig. 5d), while mutation of L534 and T538 completely abolishes the interaction with the bMERB domain (Fig. 5e, f). However, mutation of M537, Y541, and Q542 to alanine only led to minor decreases in affinity (Fig. 5g–i). The side chain of L534^CH^ inserts itself into the pocket created by L1099, R1100, M1103, and M1116 of the bMERB domain. D532, T535, and T538 of the CH domain stabilize the hydrophobic surface of the bMERB domain, which seems to be crucial for the interaction (Fig. 4d, e and Fig. 5a, b). Sequence alignments of the CH domain from EHBP1 and Mical family members show that only D532 and L534 are conserved, whereas the essential T538 is not conserved (Supplementary Fig. 4f).

Conversely, we mapped the effect of the continuous hydrophobic surface (Fig. 5b) of the full-length bMERB domain by mutating M1103, M1116, W1119, F1120, and L1099_R1100 (LR motif)^4^ to alanine. Each mutation led to a significant decrease in binding affinity with wild type CH domain (Fig. 5j–n), indicating that indeed the continuous patch of hydrophobic residues is crucial for the interaction (Fig. 5j–n and Fig. 4d, e). Interestingly, sequence alignment of the bMERB domains from EHBP1 and Mical family members shows that the LR motif and total hydrophobicity of the binding interface is quite conserved in EHBP1 and Micals. However, it appears that small changes in the amino acid composition may determine the specificity, as seen for the crucial non-conserved M1103 (Fig. 4e and Supplementary Fig. 3e).

Subsequently, we investigated whether a mutation at the CH-binding site of the bMERB domain has any effect on Rab8 binding and could show that bMERB hydrophobic mutants still form stable complexes with Rab8a with similar affinity (Supplementary Fig. 4). Since we were not able to obtain wild type bMERB:Rab8a crystals, we attempted to crystallize these mutant:Rab8a complexes, finally succeeding with the mutants of the full-length bMERB_M1116A_:Rab8a and bMERB_F1120A_:Rab8a.

### Structure of the bMERB:Rab8a complex

In order to investigate how the binding of Rab8a releases the CH domain from the bMERB domain, we aimed to determine the structure of a full-length bMERB domain in complex with Rab8a. We were able to solve the structures of the bMERB_M1116A_:Rab8a and bMERB_F1120A_:Rab8a complexes to resolutions of 1.914 Å and 2.0 Å, respectively, as described in materials and methods. The complex structures are quite similar, and we describe the bMERB_M1116A_:Rab8a structure in detail.

Two copies with an overall similar architecture of bMERB_M1116A_:Rab8a complex per asymmetric unit were observed (Supplementary Fig. 5a, b). Consistent with previously reported bMERB structures, the EHBP1 bMERB domain displays the same three helical–fold organization^4,24^ and helices 1–2 of the bMERB_M1116A_ domain have a slightly different conformation in both copies, indicating that this part is somewhat flexible (Supplementary Fig. 5b); however, the Rab8a binding site adopts the same conformation in both copies. The EHBP1_bMERB_ domain shows an RMSD of 2.3 Å for 122 residues to Mical cL_bMERB_ (PDB 5SZI), RMSD of 2.5 Å for 120 residues to Mical3_bMERB_ (PDB 5SZG) and RMSD of 3.6 Å for 119 residues to Mical1_bMERB_ (PDB 5LPN), whereas Rab8a is quite similar to Rab8a/10 of Mical cL_bMERB_:Rab8a /Mical1_bMERB_:Rab10 complex (RMSD 0.6 Å) (Supplementary Fig. 5c–e).

**Fig. 6:**
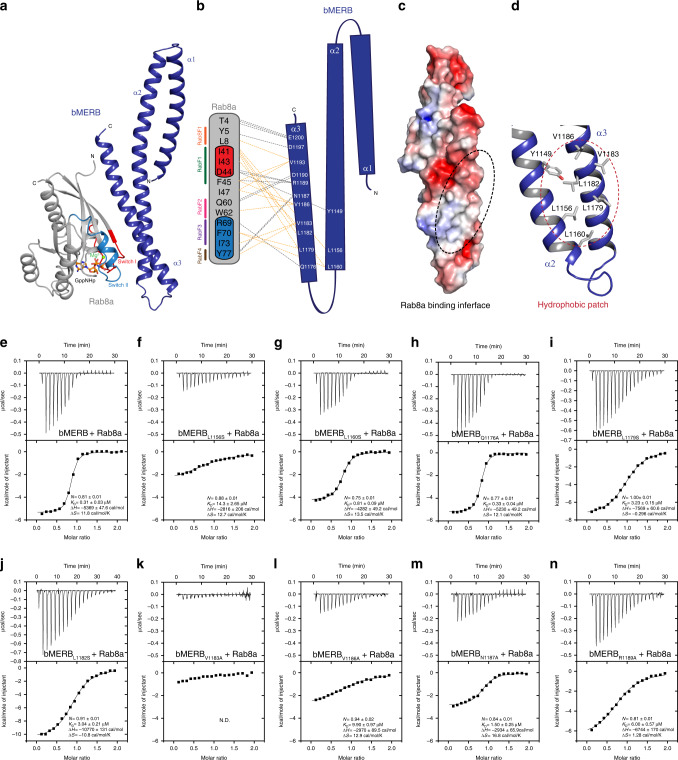
The C-terminal hydrophobic patch of the bMERB domain is crucial for Rab8a interaction. **a** Cartoon depiction of the EHBP1 bMERB_M1116A_:Rab8a_GppNHp_ complex. Rab8a_GppNHp_ (gray, chain B) binds to EHBP1 bMERB (blue, chain D) via its N-terminal regions, the switch regions as well as the inter-switch region. Switch I and switch II are shown in red and blue, respectively. GppNHp and Mg^2+^ are depicted as sticks and a green sphere, respectively. **b** Schematic illustration of the interactions between the bMERB domain and Rab8a_GppNHp_. Hydrogen bonds and ionic interactions are shown in gray dashed lines and light orange dashed lines indicate hydrophobic interactions. RabSF1, RabF1, RabF2, RabF3, and RabF4 motifs are shown in orange, green, pink, purple, and brown respectively. **c** Electrostatic potential of the bMERB domain calculated in PyMOL using the APBS-PDB2PQR plugin and visualized in red to blue (−5 kT/e to +5 kT/e). The dashed line highlights the region that interacts with Rab8a. **d** The C-terminal hydrophobic patch of the EHBP1 bMERB domain. **e**–**n** Mutational characterization of the bMERB:Rab8a complex interface. Binding of GppNHp Rab8a_1-176_ with different EHBP1 bMERB mutants was systematically tested and affinities were measured by ITC experiments. The data are representative of at least three repetitions. N.D. denotes not detected.

Similar to previously reported bMERB:Rab complex structures^4^, the major interactions between Rab8a and bMERB^EHBP1^ involve α-helix 3, and some additional interaction surface is provided by residues from α-helix 2. Hydrophobic side chains of Rab8a switch I (I41 and I43) and switch II (F70, I73, and Y77) are buried in a hydrophobic core of the interface formed by α-helices 2–3 (Y1149, L1156, L1160, L1179, L1182, and V1183) (Fig. 6c, d). A conserved triad of aromatic amino acids (F45, W62, and Y77) also forms hydrophobic interactions with V1186 and L8, F45, and I47 of Rab8a interact with V1193 of the bMERB domain (Fig. 6b and Supplementary Fig. 5b). Besides these interactions, the inter-switch region (F45, I47, Q60, and W62) and Rab subfamily motif1 (RabSF1, Y5, and L8)^33^ of Rab8 also interact with the bMERB^EHBP1^ domain. Several polar interactions were observed between Rab8a and the bMERB domain, including between side chains of T4^Rab8a^-E1200, Y5^Rab8a^-D1197, D44^Rab8a^-R1189, D44^Rab8a^-Y1149, Q60^Rab8a^-D1190, R69^Rab8a^-Q1176, Y77^Rab8a^-N1187, and the F45^Rab8a^ backbone carbonyl forms a hydrogen bond with R1189 side chain (Fig. 6a–d). T72^Rab8a^ is not involved in any direct interaction with the bMERB domain, explaining why mutant T72E has no effect on bMERB^EHBP1^ binding (Supplementary Fig. 5b).

### bMERB Ct-hydrophobic patch is crucial for Rab8 interaction

Previously, we have shown that the N-terminus of Rab8 family members provides specificity with respect to interaction with bMERB^4^. Here, we sought to determine the contribution of essential bMERB residues that are necessary for Rab8 interaction. Similar to the CH-binding site, a robust cluster of hydrophobic residues was observed in the bMERB:Rab-binding interface (Fig. 6a–d) and to test the importance of this hydrophobic patch, we mutated the leucine residues of bMERB to serine and valine to alanine (Fig. 6c, d). The L1156S mutation leads to a 47-fold reduction in affinity (Fig. 6f); L1156 is part of the LR motif that is conserved in bMERB family members (Supplementary Fig. 3e). L1156 has only one close hydrophobic contact, with I41 of Rab8a. It nevertheless appears to be essential for maintaining the integrity of the hydrophobic patch formed by helices 2 and 3 of the bMERB domain. The effect of L1160S on Rab binding was less pronounced, leading to a 2–3-fold reduction in binding affinity (Fig. 6g). However, mutations L1179S and L1182S led to a tenfold decrease in binding affinity (Fig. 6i–j). We could not detect binding in the case of the V1183A mutant that is involved in hydrophobic interactions with I43, I73, and Y77 (Fig. 6k). Altogether, our data suggest that the continuous C-terminal hydrophobic patch on bMERB is essential for the formation and stability of the Rab-binding site. Besides the hydrophobic patch residues, we also mutated several polar residues of the bMERB domain to alanine. Mutation of Q1176A does not have any effect (Fig. 6h). V1186A displays a more than 30-fold reduction in binding affinity (Fig. 6l) and this residue forms hydrophobic interactions with a conserved triad of aromatic amino acids (F45, W62, and Y77). Mutation of N1187A and R1189A led to a 5 and a 20-fold reduction in binding affinity, respectively (Fig. 6m, n). R1189 is conserved throughout the bMERB family members (Supplementary Fig. 5e). Taken together, our data suggest that the C-terminal hydrophobic patch is crucial for the Rab interaction, and the conserved arginine residue R1189 also contributes significantly to the binding affinity.

In line with the ITC results, bMERB constructs having low Rab8a binding affinity failed to form stable complexes in gel filtration experiments (Supplementary Fig. 7).

### Structural basis of CH domain release upon Rab8 binding

**Fig. 7:**
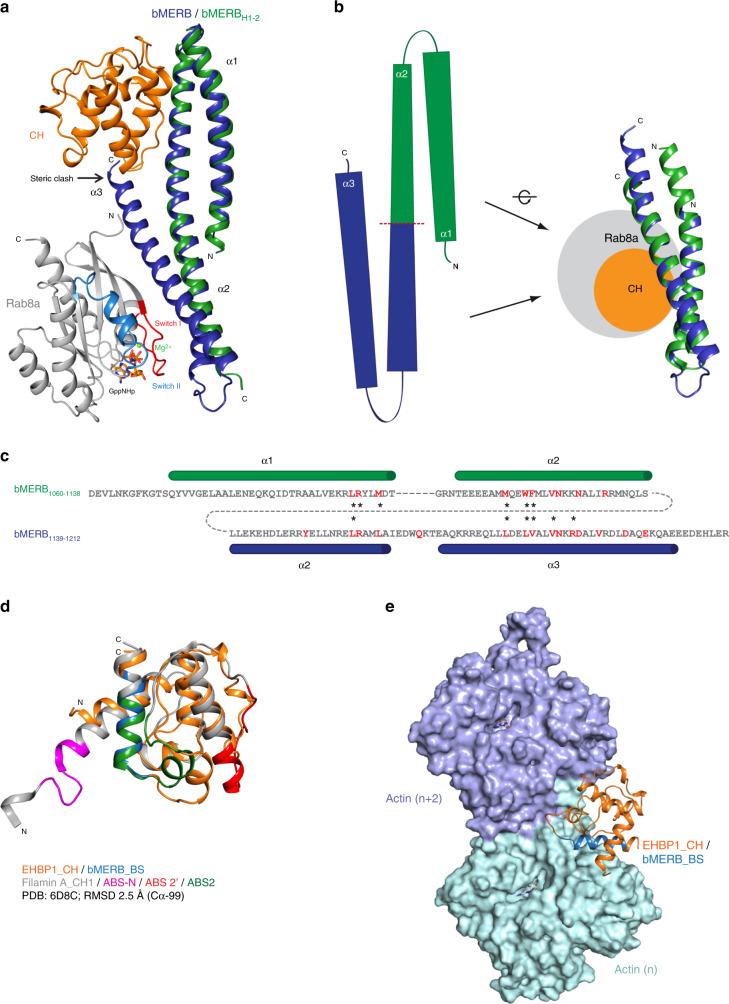
Structural basis of the CH domain release from the bMERB domain upon Rab8a binding. **a** Structural superposition of the EHBP1 bMERB_H1-2_:CH domain and EHBP1 bMERB_M1116A_:GppNHp Rab8a_1-176_ complexes. **b**, **c** Schematic presentation of the CH (green, N-terminal) and Rab8 (blue, C-terminal) binding site of EHBP1 bMERB domain. Structural and sequence alignment of the N-and C-terminal halves of the EHBP1 bMERB domain, showing strong conservation between both CH and Rab8a binding sites. CH and Rab8a interacting residues are shown in red. * denotes critical residues for the interaction. **d** Structural alignment of the EHBP1 CH domain (orange, bMERB binding site (bMERB_BS) in blue) with the filamin A CH1 domain (gray). F-actin binding sites in the filamin A CH1 domain are shown in pink (ABS-N), red (ABS2´), and in green (ABS2). (ABS actin binding site). **e** Structural model of the F-actin:EHBP1 CH domain complex, based on the complex structure of filamin A CH1 domain:F-actin (PDB ID 6D8C). The bMERB binding site (bMERB_BS, blue) of the CH domain is buried by the actin filament.

To unravel the structural basis for the release of the CH domain from the bMERB domain upon Rab8 binding, we superimposed the bMERBH_1-2_:CH complex structure with that of bMERB_M1116A_:Rab8a (Fig. 7a). The first two helices of the bMERB domain adopt the same conformation in both structures, while the third helix of the bMERB domain in the presence of Rab8 adopts a defined conformation which would infringe spatially on the CH domain binding site, suggesting that the CH:bMERB:Rab8 triple complex could not be formed because of a steric clash (Fig. 7a and Supplementary Fig. 8a). This finding complements our transient kinetic data showing that the binding of Rab8a to the bMERB domain releases the CH domain. The structures also suggest that in the absence of Rab8a, helix 3 of bMERB domain must adopt a different conformation, or be flexible, so that the full-length bMERB domain can form a stable complex with the CH domain.

To compare the Rab-and CH-binding sites on the bMERB domain, we have cut the second helix in the middle and aligned the resulting two hairpins (Fig. 7b). This alignment shows that the two halves of the bMERB domain are quite similar, a characteristic feature of bMERB family members indicating that bMERB domains are evolved by gene duplication of this helical hairpin^4,5^. The sequence alignment shows that the residues that are involved in CH and Rab binding are quite similar and mutation of some of these residues led to similar effects (Fig. 7c).

Further, to understand the biological significance of CH domain release and to identify the probable F-actin binding site in the CH domain, we have utilized the filamin A ABD_E254K:F-actin (PDB 6D8C, 3.6 Å) cryo-EM structure^34^. A superposition of the CH1^FLNa^ and CH^EHBP1^ domains indicates a similar structure (RMSD of 2.5 Å, 99 aligned Cαs) (Fig. 7d and Supplementary Fig. 8b–d). To understand the binding mode, the CH^EHBP1^ domain was superimposed onto the CH1^FLNa^ domain. To optimize the contacts to the actin filament and to obtain the best fit to the CH-binding pocket, the CH^EHBP1^ model was manually adjusted to relieve minor clashes, mainly by shifting the short helix 522–529 (residues PSDMVLLA) and the following loop, and then energy minimized with Macromodel (Schrodinger suite^35^) and PHENIX^36^. It is clear that the main contact site of the CH^EHBP1^ domain that corresponds to ABS2 is occupied by bMERB in the CH:bMERB complex structure, indicating that the bMERB domain has to dissociate to facilitate F-actin binding of the CH domain. The optimized CH^EHBP1^:F-actin model has a smaller buried interaction interface area (4640 Å^2^) between the CH domain and F-actin compared with the CH1^FLNa^:F-actin (6000 Å^2^) (Fig. 7e). This is mainly due to missing ABS-N and a smaller ABS2´ in the CH^EHBP1^ domain (Fig. 7d and Supplementary Fig. 7c). Since the CH^EHBP1^ domain has lower affinity to F-actin compared with filamin A, the smaller buried interface could indeed correspond to the physiological situation. However, we cannot rule out the possibility of different or more extensive F-actin induced conformational changes in the CH^EHBP1^ domain upon binding. Further structural studies are required to understand the F-actin:CH^EHBP1^ interaction. However, our model suggests that for F-actin association, the CH^EHBP1^ domain has to be free, i.e., not bound to the bMERB domain.

## Discussion

EHBP1 has a central role in vesicular trafficking and lipophagy, yet its regulation is not understood. This work offers mechanistic insight into the EHBP1 activation mechanism by Rab8 family members. We have shown that both N-and C-termini of EHBP1 have the potential to interact with the membrane; the NT-C2 domain binds to PI(3)P, PI(5)P, and phosphatidylserine, lipid molecules present in the early endosomes and plasma membrane respectively. Previously, we reported that the EHBP1 CaaX box can be farnesylated^4^. The presence of two membrane-associating moieties can fine-tune the EHBP1 function by regulating its localization.

We have found that unlike Mical1, EHBP1 has only a single highly conserved C-terminal high affinity Rab-binding site and the so-called low-affinity binding site can be occupied by the CH domain. In the absence of Rab8, EHBP1 exists in a closed form with the C-terminal bMERB domain interacting with the central CH domain so that its binding with actin filaments is perturbed. Several previous studies indicated that an intramolecular interaction occurs between the bMERB domain with the various other domains (CH/LIM/MO-CH-LIM), and were suggested to be inhibitory^21–24^. Fremont et al., proposed a model for Mical1 activation in which an intramolecular association occurs between the C-terminal bMERB domain and the N-terminal mono-oxygenase together with the CH and LIM domains (MO-CH-LIM), forming an enzymatically dead complex. Binding of Rab35 can release the auto-inhibition, resulting in increased F-actin depolymerization^19,20^. Mical-like family members also engage in intramolecular interaction between their N-terminal CH domain (Mical-L1) or LIM domain (Mical-L2) with their C-terminal bMERB domain^21,23^. The present work is the first to perform biochemical characterization of the auto-inhibited bMERB complexes, but, we could not detect any interaction between the bMERB domain of Mical family members with their respective CH/LIM/CH-LIM domain (Supplementary Fig. 2), although this could be due to a weaker affinity, which is high enough to lead to an intramolecular interaction, but not enough to be detected in the intermolecular situation. Further experiments with longer constructs are required.

Here, we report the CH:bMERB and bMERB:Rab8 complex structures and demonstrate that the integrity of both N-and C-terminal hydrophobic patches is crucial for the CH and Rab interactions. We show that the CH and Rab-binding sites of the bMERB domain have significant similarities (e.g., the conserved LR motif of helix 1^CH_bs^ and helix2^Rab_bs^ is essential for CH/Rab8 interaction and similarly CH and Rab aligned interacting residues M1116/L1179, W1119/L1182, and F1120/V1183 are for the CH/Rab interaction). Small changes in the amino acid sequence of the two halves determine the specificity toward CH or Rab binding.

Structural alignment of the EHBP1_bMERB_:Rab8 complex with other published structures shows that the C-terminal high affinity Rab-binding site is quite conserved in all structures (Supplementary Fig. 5c–e). However, the conformation of helix 1 and helix 2 differs at the CH/^2nd^Rab-binding site. Further, superposition of the Mical1_bMERB_:Rab10 (1:2) complex with EHBP1_bMERB_M1116A_:Rab8a shows that the conformation of EHBP1 helix 1 and helix 2 clashes with the 2nd Rab10 molecule. For effectors displaying a 1:2 stoichiometry with Rab8 (like Mical 1), we have previously proposed a model where the two Rab-binding sites have separate functions, with the first Rab binding leading to membrane recruitment, and subsequently to the release of auto-inhibition of the CH/LIM domains by binding of the second Rab^4^. In contrast, EHBP1 appears to have the intrinsic ability to be targeted to the membrane, and Rab8 only binds to the highly conserved C-terminal Rab-binding site of the bMERB domain, changing helix 3 conformation, and leading to release of auto-inhibition.

In agreement with recent work on Mical1^37^, we show that phosphomimetic mutation of the switch II threonine of Rab8/10 does not affect its interaction with the EHBP1_bMERB_ domain and is not part of the bMERB binding interface (Supplementary Fig. 5b). In contrast, a study of the RILPL2:Rab8 interaction has shown that phosphorylation of switch II T72 is essential for the interaction and also stabilizes the RILPL2:Rab8 complex (PDB 6RIR)^38^, suggesting that the phosphorylation of switch II threonine has a selective effect on different effector molecule interactions.

**Fig. 8:**
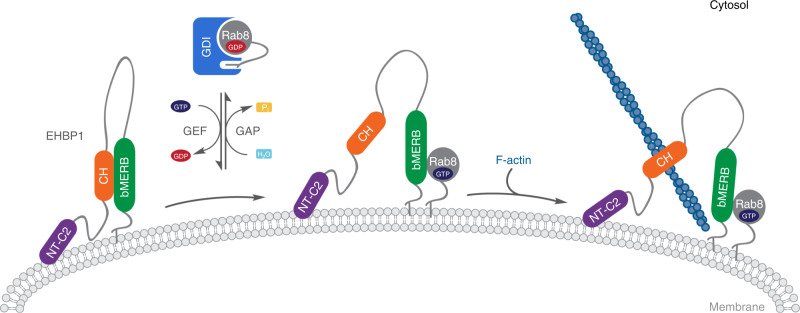
Mechanistic model of the EHBP1 activation by Rab8 family members. In the absence of active Rab8, EHBP1 exists in a closed conformation in which the CH domain interacts with its bMERB domain. Rab8 is recruited to the membrane by its GEF molecule (e.g., Rabin 8 or GRAB) and the resulting active GTP bound Rab8 interacts with the EHBP1 bMERB domain; this interaction makes the CH domain available for F-actin interaction.

In the last part of the work, we have shown that unlike a conventional F-actin binding domain, which requires an open conformation CH1-CH2 structure, the CH^EHBP1^ (CH2-type) domain binds to F-actin^25,26^. Previously, it was shown that the *C. elegans* CH^EHBP1^ domain directly interacts with F-actin, and the authors suggested that Rab10 binding to the bMERB domain enhances the CH domain:F-actin interaction^7^. However, no difference was detected in the F-actin binding of the free CH domain versus a CH-bMERB fusion construct^7^. The authors did not consider an auto-inhibition model; instead, they proposed a model where Rab10 binding leads to multimerization of EHBP1, leading to a side by side placement of CH domains from the dimeric EHBP1, but no experimental proof is provided. Recently, Miyake et al. have reported a closed conformation mouse Mical-L1 (LIM:bMERB) and binding of Rab13 opens up the Mical-L1, allowing its interaction with F-actin^23^. By homology modeling, the authors built the LIM:bMERB:Rab13 tripartite complex and also docked the LIM domain onto F-actin and suggested that for the F-actin interaction, the LIM domain has to be free. However, this model could not explain how Rab13 binding to the bMERB domain releases the LIM domain. They suggested that a second competitive Rab13 binding at the N-terminal binding site is a prerequisite for the LIM:F-actin interaction. No stoichiometry is reported for the Mical-L1_bMERB_:Rab13 complex, and appropriate biochemical and structural studies would be needed to characterize the bMERB:Rab13 and LIM:bMERB complexes. In the current study, we clearly showed that the binding of Rab8 to bMERB releases the CH domain and further, our CH:F-actin model suggests that for F-actin interaction, the CH domain has to be free, since the bMERB-binding site is also part of the F-actin binding site (Fig. 8).

## Methods

### Plasmid cloning

Prokaryotic and eukaryotic expression constructs were generated by standard cloning techniques, using Phusion polymerase, restriction digestion, and ligation by T4 DNA ligase. Point mutants were generated by quick-change site-directed mutagenesis, using Phusion polymerase. A detailed overview of all expression vectors employed in this study is presented in Supplementary Table 2. Primers used for plasmid cloning are listed in Supplementary Table 3. All plasmids were verified by DNA sequencing.

### Fluorescence microscopy

The full length human mCherry-tagged Rab constructs (Rab8a_Q67L_, Rab10_Q68L_) used in this paper were described previously^4^. Full-length human EHBP1_isofrom3_, ΔNT-C2-EHBP1_isofrom3_, and EHBP1_isofrom3_ΔCaaX constructs were cloned into the pEGFP (C1) vector between XhoI and SmaI sites by conventional PCR using Human EHBP1 cDNA (Isoform 3, Dharmacon). The NT-C2 domain was cloned into the pEGFP (N1) vector between XhoI and SmaI sites and all constructs were verified by DNA sequencing. Cos7 cells (ATCC: CRL-1651) were maintained in DMEM medium supplemented with 10% fetal bovine serum, 2 mM L-glutamine, and penicillin/streptomycin at 37 °C in the presence of 5% CO_2_. Cells were grown on a coverslip in 6-well plates until they reached 60–70% confluency and transiently transfected using linear polyethylenimine, MW 25000 (PEI, Polysciences Inc, 3:1 PEI:DNA (12:4 µg). Expression was checked 16–24 h post transfection. Cells were fixed with 3.7% paraformaldehyde in PBS for 15 min at room temperature. After washing with PBS, coverslips were mounted on glass slides with SlowFade Gold antifade reagent (Invitrogen). For EHBP1 constructs, single-plane images were taken by an EVOS fl fluorescence microscope equipped with 60x/1.42 Plan-Apochromat oil immersion objective.

EHBP1:Rab co-localization images were taken with a Zeiss LSM800 confocal microscope equipped with a Plan-Apochromat 63x/1.4 Oil DIC M27 oil immersion objective. 3D stacks of 0.37 μm steps were acquired and images from all focal planes were rendered as a single maximum-intensity projection image using ImageJ software and later assembled with Adobe Illustrator.

### Lipid overlay assay

To assess the lipid-binding properties of the EHBP1 NT-C2 domain, we performed a protein-lipid overlay assay with recombinant His6-MBP (MBP: maltose binding protein)-NT-C2 domain fusion protein and His6-MBP was used as a control. 1 ng of His6-MBP-NT-C2 domain or His6-MBP as also blotted on the top of the PIP strip (Echelon Biosciences) to serve as a positive control (Antibody control). Strips were blocked for 1 h at RT in 3% (w/v) fatty acid-free BSA (Sigma) in TBST (50 mM Tris-HCl pH 7.5, 150 mM NaCl, and 0.1% (v/v) Tween 20). PIP strips were then incubated overnight at 4 °C with gentle mixing in the blocking solution containing either 1 µg/ml of purified His6-MBP-NT-C2 domain or His6-MBP proteins. 1 mM of CaCl_2_ was added to the control and two one of the strip while in the second strip, 5 mM EGTA was added. The membranes were later incubated for 1 h with a 1:2000 dilution of anti-His antibody (Sigma) followed by three 10 min washing steps with TBST, and strips were then incubated with HRP goat anti-mouse antibody (Cayman chemicals) with a 1:5000 dilution. The washing steps were repeated and following development using the SuperSignal West Dura Substrate (Thermo Scientific) images were taken with a GelDoc system (Bio-Rad).

### Recombinant protein expression and purification

Human Rab G-domains (Rab8a_1-176_ and Rab10_1-175_) and phosphomimetic Rab (Rab8a_1-176_T72E_ and Rab10_1-175_T73E_) were expressed and purified as described previously^39^. Rabs were preparatively loaded with GppNHp (Guanosine-5′-[β-γ-Imido]-triphosphate) or mant GppNHp (2′/3′-O-N-Methyl-anthraniloyl)-guanosine-5′-[(β,γ)-imido]triphosphate) and the reaction was performed as described previously^4^. Nucleotide exchange efficiency was quantified by C18 reversed-phase column (Prontosil C18, Bischhoff Chromatography) with HPLC in 50 mM potassium phosphate buffer pH 6.6, 10 mM tetrabutylammonium bromide, and 12% acetonitrile (v/v) and for the mant GppNHp exchange run the buffer contains 25% acetonitrile (v/v). Protein samples were heat precipitated at 95 °C for 5 min and centrifuged at 15,700 g for 10 min and loaded (25 µM, 20 µl) on the column. Peaks were integrated and to determine the nucleotide retention times, a nucleotide standard run was performed.

All other proteins were recombinantly expressed in *E. coli* BL21 DE3-RIL (Agilent) cells in LB media supplemented with proper antibiotics and cells were grown at 37 °C to OD_600_ nm = 0.8–1.0 and stored at 4 °C for 30 min. Further expression was induced by the addition of 0.5 mM IPTG, and cells were allowed to grow at 20 °C for 14–16 h. Cells were pelleted and stored at −80 °C until ready for purification. Cells were mechanically lysed by passing through a fluidizer (Microfluidic**)** in a buffer [50 mM Hepes pH 8.0, 500 mM NaCl or LiCl, 2 mM βME (2-Mercaptoethanol)] and 1 mM PMSF (Phenylmethylsulfonyl fluoride) and lysates were cleared by centrifugation at 75,600 g for 30 min. Subsequently, the proteins were purified by Ni^2+^-affinity chromatography (HiTrap, GE Healthcare). For the His6-MBP-NT-C2 domain purification, the protein was concentrated after first Ni^2+^-affinity chromatography and subjected to gel filtration (Superdex 75 26/60, GE Healthcare) in the final buffer (20 mM Hepes 7.5, 200 mM NaCl and 2 mM DTE).

For the human EHBP1 CH, bMERB domain and other protein purifications, cells were lysed in a buffer (50 mM Hepes pH 8.0, 500 mM NaCl or LiCl and 2 mM βME (2-Mercaptoethanol) having 1% CHAPS and lysates were cleared by centrifugation at 25,000 rpm for 30 min. Subsequently, the proteins were purified by Ni^2+^ affinity chromatography (HiTrap, GE Healthcare). The His6-tag was cleaved by Tobacco Etch Virus (TEV)-protease, and a second Ni^2+^-affinity purification was performed to remove the TEV protease and His6-tag. The final purification step was achieved by gel filtration (Superdex 75 26/60, GE Healthcare) (final buffer: 20 mM Hepes 7.5 or 8.0, 100 mM NaCl and 2 mM DTE). The purified protein was collected and concentrated; flash-frozen in liquid N_2_, and stored at −80 °C.

### Analytical size exclusion chromatography

The bMERB:GppNHp Rab8a_1-176_ complex formation was analyzed by aSEC. 110 µM of bMERB domain and 121 µM of GppNHp Rab8_1-176_ protein (Effector: Rab stoichiometry of 1:1.1) was mixed in a buffer containing 20 mM Hepes 7.5, 50 mM NaCl, 1 mM MgCl_2_ and 2 mM DTE (Rab buffer) and centrifuged for 15 min at 15,700 g at 4 °C. 40 µl of the mixture was injected into a Superdex 75 10/300 GL gel filtration column (GE Healthcare) pre-equilibrated with the Rab buffer with a flow rate of 0.5 ml/min at room temperature and absorption at 280 nm was recorded.

Similarly, the complex formation between the bMERB (110 µM) domain and the CH (121 µM) domain was analyzed in the buffer containing 20 mM Hepes 7.5, 150 mM NaCl, and 2 mM DTE (CH buffer) and centrifuged for 15 min at 15700 g at 4 °C. 40 µl of the mixture was injected onto a Superdex 75 10/300 GL gel filtration column (GE Healthcare) pre-equilibrated with the CH buffer with a flow rate of 0.5 ml/min at room temperature and absorption at 280 nm was recorded.

### Isothermal titration calorimetry

Protein–protein interaction measurements were conducted by ITC using an ITC200 microcalorimeter (MicroCal). bMERB:Rab interaction measurements were performed in buffer containing 20 mM Hepes 7.5, 50 mM NaCl, 1 mM MgCl_2_, and 1 mM Tris (2-carboxymethyl) phosphine (TCEP) whereas bMERB: CH interactions were performed in buffer containing 20 mM Hepes 7.5, 150 mM NaCl and 1 mM TCEP at 25 °C. Wild types and mutant proteins were dialyzed overnight in their respective buffer. Samples were centrifuged at 15,700 g for 30 min at 4 °C and protein concentration was determined by Bradford assay (Bio-Rad). 500 µM of GppNHp Rab8a_1-176_ was titrated into the cell containing 50 µM bMERB domain and for bMERB:CH interaction 600–800 µM of CH domain was titrated into the cell containing 40–60 µM bMERB domain. For the control experiments, the buffer was titrated into the cell containing the bMERB domain and in the second control experiment, the CH domain was titrated against buffer. The binding isotherms were integrated and the data were fitted to a one-site-binding model using Origin 7.0 (MicroCal). The reported ITC result is the representative one of at least three independent measurements.

### Cy3 labeling of the CH domain

100 µM the CH domain is incubated with 300 µM Cy3-thioester (Jena bioscience) in the buffer containing 50 mM Hepes pH 7.5, 150 mM NaCl and 50 mM MPAA (4-Mercaptophenylacetic acid, Merck) at RT for 1 h. Free dye was removed by passing through a PD10 column (GE Healthcare) in buffer containing 50 mM Hepes pH 7.5, 150 mM NaCl, and 2 mM DTE. To check the extent of labeling, samples were analyzed by mass spectrometry (LC/ESI-MS) (Supplementary Fig. 2k).

### Transient kinetic measurements

bMERB: Rab kinetic measurements were performed in Rab buffer (20 mM Hepes 7.5, 50 mM NaCl, 1 mM MgCl_2_, and 2 mM DTE) using a SX-20 stopped-flow apparatus (Applied Photophysics) at 25 °C. For bMERB:mantGppNHp Rab8a_1-176_ kinetic measurements, the experiments were performed using the signal from the methylanthraniloyl group of mantGppNHp, the mant group was excited with a 360 nm LED, and emission was detected through a 420 nm cutoff filter.

For bMERB:CH kinetic measurement, the experiments were performed in CH buffer using the signal from the Cy3 group of the N-terminal Cy3 labeled CH domain and was excited with a 535 nm LED and emission was detected through a 570 nm cutoff filter. For Rab8a dissociation experiments, CH buffer (20 mM Hepes 7.5, 150 mM NaCl, and 2 mM DTE) was used. All stopped-flow results that were analyzed are averages of 6–8 individual traces. Single exponential functions were fit using the Origin9 software (OriginLab).

### Crystallization and structure determination

Initial crystallization condition screens for all protein complexes described in the paper were performed with the JSG Core I-IV, Pact, and Protein Complex suites (Qiagen). The sitting-drop vapor diffusion method was used, with a reservoir volume of 70 μl and a drop volume of 0.1 μl protein (300–400 µM complexes, 1:1 Rab:effector) and 0.1 μl reservoir solution at 20 °C. The best conditions were then optimized using the sitting-drop vapor diffusion method varying drop sizes in order to obtain well diffracting crystals. The complex of bMERB_H1-2_:CH (300 µM of 1:1 complex) was crystallized in 0.18 M Tri-ammonium citrate and 20% (w/v) PEG 3350. The complex of bMERB_M1116A_:Rab8a_1-176_GppNHp_ (400 µM of 1:1 complex) was crystallized in 0.1 Mes pH 6.0, 5% (w/v) PEG 3000, and 30% (v/v) PEG 200. The complex of bMERB_F1120A_:Rab8_1-176_GppNHp_ was crystallized in 0.1 Mes pH 6.5, 10% (w/v) PEG-MME 5000 and 12% (v/v) 1-Propanol. Crystals were fished directly from the crystallization drop and flash-cooled in liquid nitrogen. Diffraction data were collected at 100 K on beamline X10SA at the Swiss Light Source (Paul Scherrer Institute, Villigen, Switzerland). For the bMERB_H1-2_:CH complex crystal, a native data set was collected at a wavelength of 1.000010 Å whereas two data set from a single crystal was taken for the bMERB_M1116A_:Rab8a_1-176_GppNHp_ complex at a wavelength of 0.919532 Å. A native data set was collected for bMERB_F1120A_:Rab8a_1-176_GppNHp_ complex at a wavelength of 0.919550 Å. Data were integrated and scaled with XDS^40^.

The Crystal of bMERB_H1-2_:CH complex diffracted to a resolution of 2.2 Å (space group P2_1_ with a = 54.06 Å, b = 48.19 Å, c = 100.30 Å) and two copies of the complex is present in the asymmetric unit of the crystal. The initial model for bMERB_H1-2_:CH complex was obtained by molecular replacement using PHASER^41^ with the NMR structure of the EHBP1 CH domain (PDB 2DK9) as a search model^32^. The partial model was completed with PHENIX AutoBuild^42^ and manual building in Coot^43^. For the bMERB_M1116A_:Rab8a_1-176_GppNHp_ complex, the crystal diffracted to a resolution of 1.914 Å (space group C2 with a = 116.36 Å, b = 35.38 Å, c = 165.67 Å) and two copies of the complex constitute the asymmetric unit of the crystal. The partial model was obtained by MR using PHASER^41^ and the Rab8a (PDB 5SZI) was used as a search model^4^. The initial model was completed with PHENIX AutoBuild^42^ and by manual building in Coot^43^. The final models were refined to convergence with phenix.refine^36^ or Refmac5^44^ of the CCP4 package^45^. The final model of the bMERB_M1116A_:Rab8_1-176_GppNHp_ complex was refined with phenix.refine using refined Translation/Libration/Screw tensors, which further lowered the Rfree by 9%. For the bMERB_F1120A_:Rab8a_1-176_GppNHp_ complex, the crystal diffracted to a resolution of 2.0 Å (space group C2 with a = 117.34 Å, b = 35.66 Å, c = 168.56 Å) and bMERB_M1116A_:Rab8a_1-176_GppNHp_ complex was used as a model for molecular replacement.

Data collection and refinement statistics are summarized in Supplementary Table 1. Structural figures were prepared using PyMOL (DeLano Scientific; http://www.pymol.org).

### Actin co-sedimentation assay

Rabbit skeletal muscle G-actin (AKL99) was purchased from Cytoskeleton and polymerized into F-actin according to the manufacturer’s protocol. F-actin (10 μM) was incubated for 1 h at RT with different concentrations of the EHBP1 CH domain (5–60 µM) in the buffer containing 5 mM Tris-HCL pH 7.5, 0.18 mM CaCl_2_, 15 mM KCl, 1 mM DTT, and 1.8 mM NaN_3_. Samples were centrifuged at 100,000 g for 1 h at 4 °C. The supernatant and pellet were subjected to 18% SDS-PAGE, followed by Coomassie Brilliant Blue staining. The quantitative analyses were performed using the Bio-Rad image analysis software in the ChemiDoc system (Bio-Rad).

For comparative actin co-sedimentation assays, 10 μM F-actin was incubated with 40 μM CH domain from EHBP1, Mical1, Mical3, and Mical-L1, and co-sedimentation was performed as described above.

### Bioinformatics

Multiple sequence alignments were generated using Clustal Omega^46^. The protein interaction interfaces from the asymmetric unit were examined in detail using the PDBePISA server (Proteins, Interfaces, Structures and Assemblies)^47^. DALI server was used for structural comparison^29^.

### Reporting summary

Further information on research design is available in the Nature Research Reporting Summary linked to this article.

## Supplementary information

Supplementary information

Peer Review File

Reporting Summary

## Data Availability

Data supporting the findings of this paper are available from the corresponding authors upon reasonable request. A reporting summary for this paper is available as a Supplementary Information file. Protein coordinates and structure factors have been submitted to the Protein Data Bank under accession codes PDB 6ZSH (bMERB_H1-2_:CH), PDB 6ZSI (bMERB_M1116A_:Rab8a), and PDB 6ZSJ (bMERB_F1120A_:Rab8a). Source data are provided with this paper.

## Source data

Source Data
