# Supplementary material for: The mechanism of activation of the actin binding protein EHBP1 by Rab8 family members
Source: Nat Commun. 2020 Aug 21;11:4187. doi: 10.1038/s41467-020-17792-3 (PMC7442826; doi:10.1038/s41467-020-17792-3)
Supplement: Supplementary file 1 — Supplementary information [file 41467_2020_17792_MOESM1_ESM.pdf]

Supplementary information for

The mechanism of activation of the actin binding protein EHBP1 by Rab8 family members

Rai *et al*.,

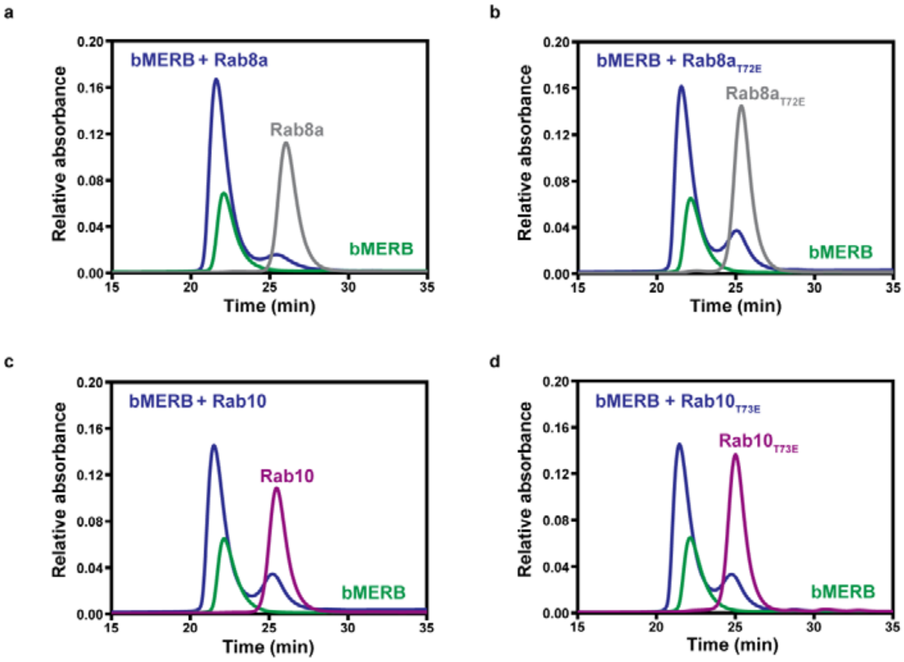

Supplementary Fig. 1: Interaction of the EHBP1 bMERB domain with Rab8a and Rab10. (**a-d**) To test for complex formation, the EHBP1 bMERB domain (green), GppNHp Rab8a$_{1\text{-}176}$ (gray)/Rab10$_{1\text{-}175}$ (magenta) and a mixture of both (blue) were loaded onto a Superdex75 10/300 GL column. Similar to wild type, phosphomimetic mutants of switch II threonine of Rab8a/Rab10 form a stable complex with the EHBP1 bMERB domain.

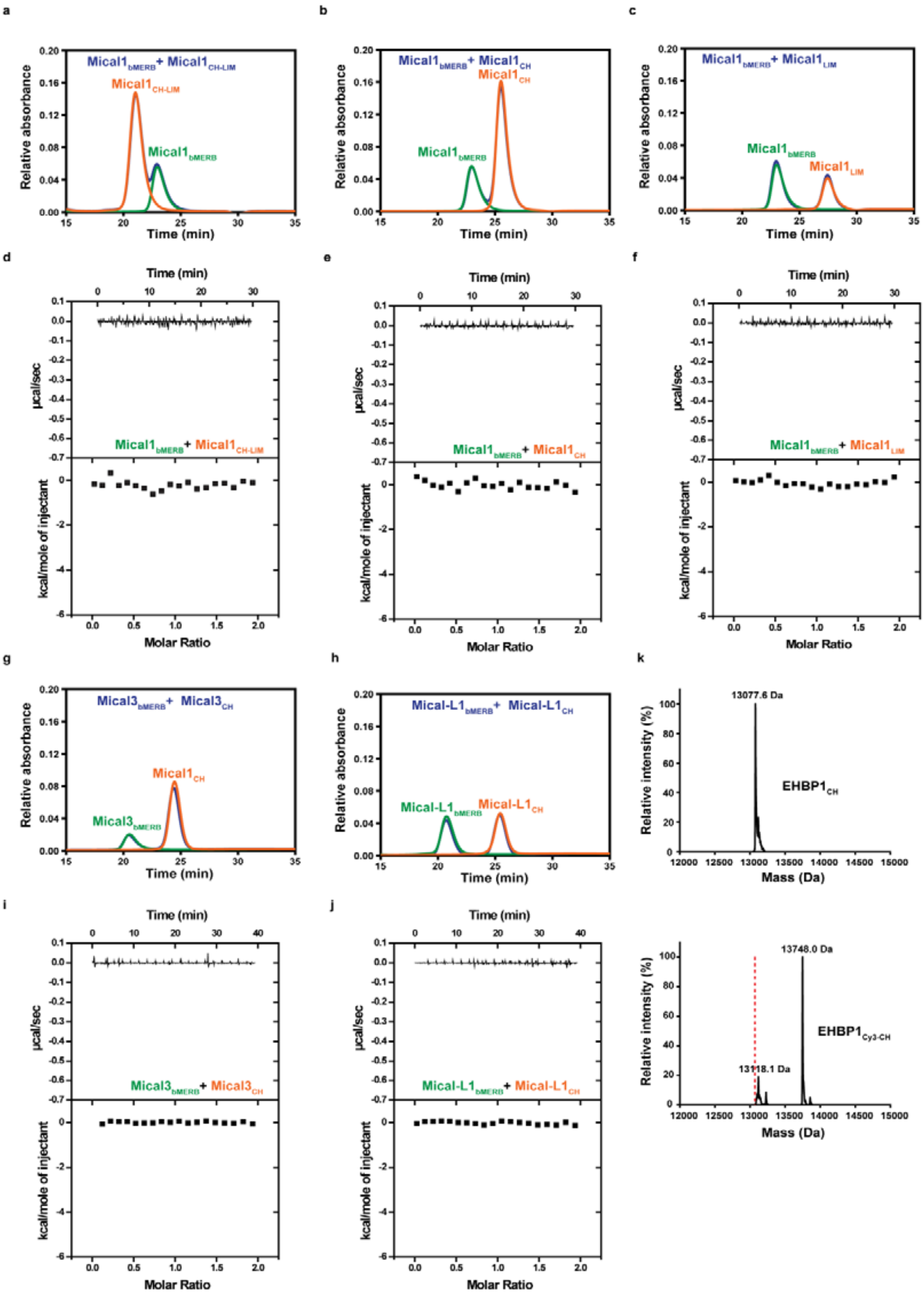

Supplementary Fig. 2: Interaction of various CH, LIM or CH-LIM domain of human bMERB family members with their bMERB domain. (**a, b, c, g and h**) The different bMERB domains (green), their corresponding CH/LIM/CH-LIM domains (orange) and mixtures of both (blue) were loaded onto a Superdex 75 10/300 GL column to test for complex formation. (**d, e, f, I and j**) ITC experiments were also performed to check for complex formation. 600 µM of CH/LIM/CH-LIM domain was titrated to 60 µM of the bMERB domain. No complex formation was observed in all combinations tested. **(k)** Cy3 labelling of EHBP1 CH domain by Cy3-thioester. The theoretical mass of the EHBP1 CH domain is 13077.1 Da and Cy3 labelling leads to an increase in the mass of ~672 Da.

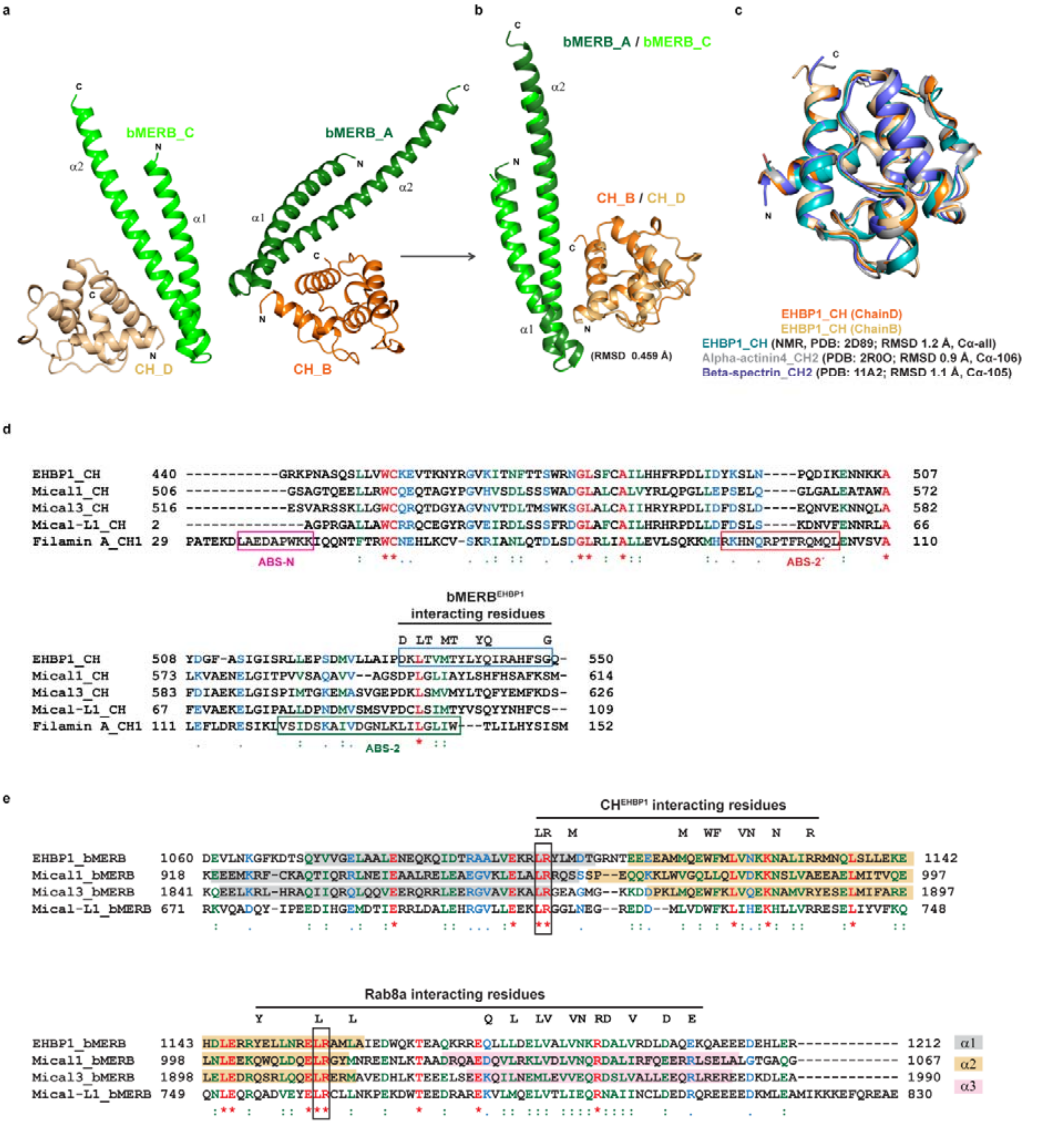

Supplementary Fig. 3: Structure of the human EHBP1 CH:bMERB complex. (**a**) The asymmetric unit of CH:bMERB$_{H1-2}$ complex is composed of two complexes of each of CH (orange/wheat) and bMERB$_{H1-2}$ domain (light green/dark green). (**b**) Overlay of both copies of the CH:bMERB complex in the asymmetric unit reveals that the interaction area and helix conformation of bMERB is conserved. (**c**) Overlay of both copies of the CH domain in the asymmetric unit with the EHBP1 CH domain NMR structure (PDB 2D89)[1], alpha-actinin4 CH2 domain (PDB 2R0O)[2] and beta-spectrin CH2 domain (PDB 1AA2)[3]. (**d**) Sequence alignment of the CH domain of different bMERB family members and the filamin A CH1 domain using Clustal Omega. F-actin binding sites of filaminA CH1 domain are shown in the boxes (pink: ABS-N, red: ABS-2´ and green: ABS2). bMERB domain interacting region of the EHBP1 CH is shown in the light blue box. Reduces involved in direct interactions with bMERB domain are shown on the top of the box. (**e**) Sequence alignment of the bMERB domain of different bMERB family members**.** Conserved LR motifs are shown in the black boxes. Residues involved in direct interactions with CH/Rab8a are shown on the top of the sequence.

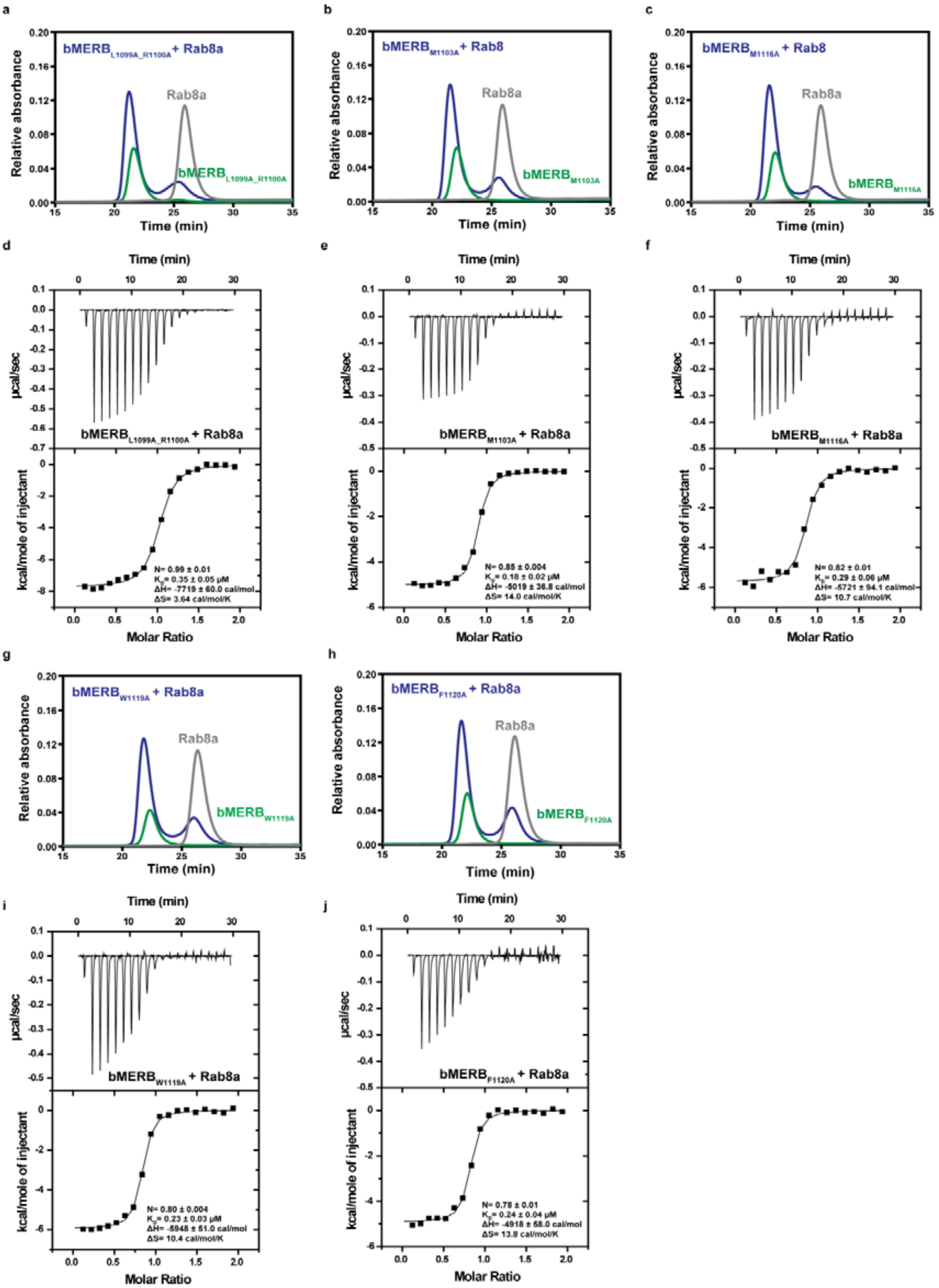

Supplementary Fig. 4: Interaction of EHBP1 bMERB domain mutants with Rab8a. (**a, b, c, g and h**) The bMERB domain mutants (green), GppNHp $Rab8_{1\text{-}176}$ (gray) and the mixture of both (blue) were loaded onto a Superdex 75 10/300 GL column. Consistently, aSEC results show that a mutation at the CH binding site of the bMERB domain does not perturb Rab8a binding. (**d, e, f, i and j**) ITC measurements show that CH-binding deficit bMERB mutants do not affect Rab8a interaction and show similar affinity.

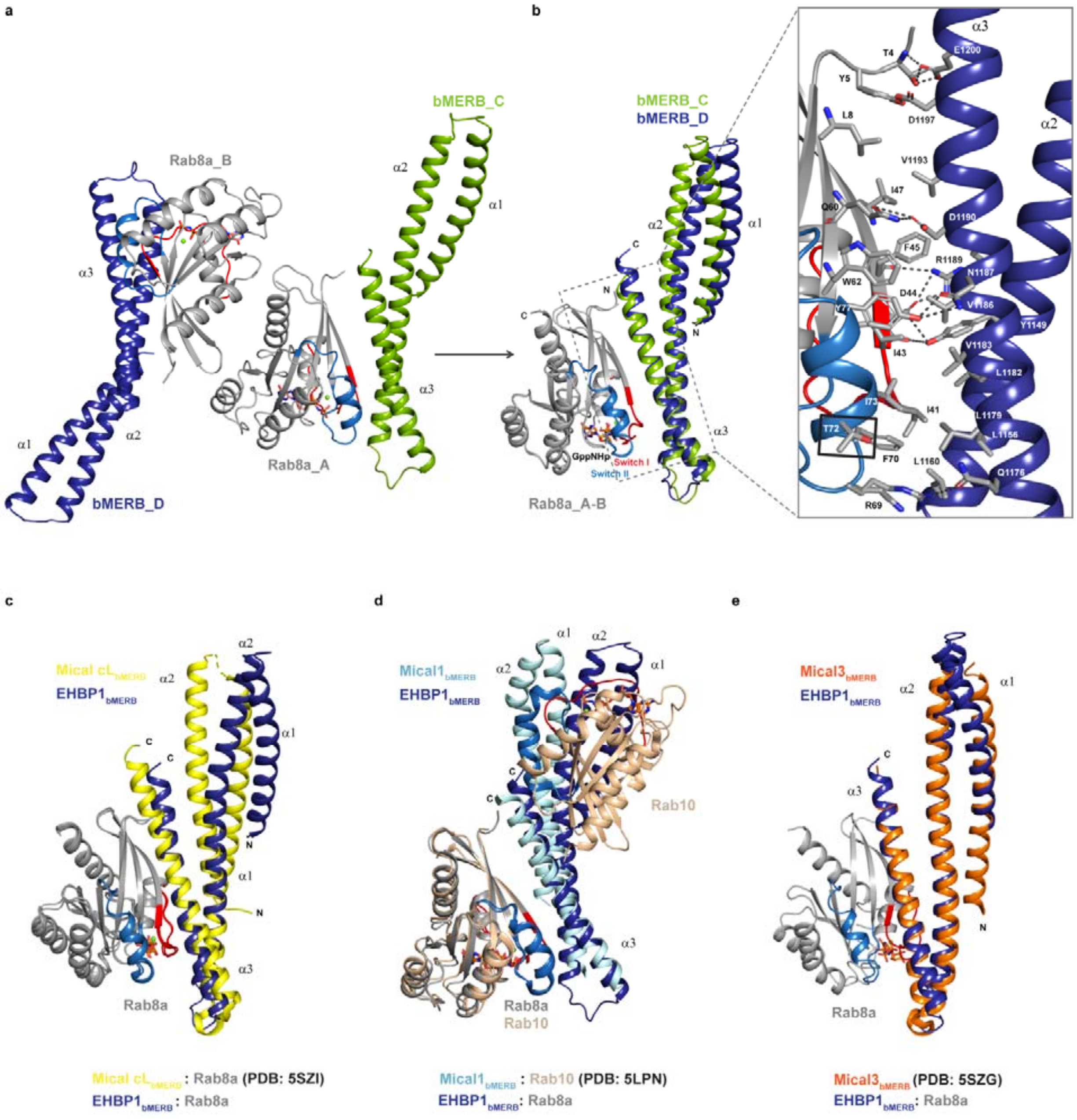

Supplementary Fig. 5: Structure of EHBP1 bMERB$_{M1116A}$:Rab8a$_{GppNHp}$ complex. (**a**) The asymmetric unit of bMERB$_{M1116A}$:Rab8$_{GppNHp}$ complex is comprised of two complexes of each of bMERB (blue/green) and Rab8a (gray). (**b**) Overlay of the both copies of bMERB$_{M1116A}$:Rab8a$_{GppNHp}$ complex in the asymmetric unit reveals that the Rab8a binding site is quite conserved; however, helix 1 and 2 adopt slightly different conformations, indicating intrinsic flexibility. The inset shows a zoomed-in overview of the interaction interface. Switch I and switch II are shown in red and blue, respectively. GppNHp and Mg^{2+} are shown as sticks and a green sphere, respectively. Hydrogen bonds and polar interactions are shown in gray dashed lines. T72 phosphorylated by LRRK2 is shown in the black box. (**c**) Structural superposition of the EHBP1 bMERB$_{M1116A}$:Rab8$_{GppNHp}$ and Mical cL$_{bMERB}$:Rab8a$_{GppNHp}$ complexes (PDB 5SZI)[4]. (**d**) Structural superposition of the EHBP1 bMERB$_{M1116A}$:Rab8a$_{GppNHp}$ and Mical1$_{bMERB}$:Rab10 complexes (PDB 5LPN)[4]. (**e**) Structural superposition of the EHBP1 bMERB$_{M1116A}$:Rab8a$_{GppNHp}$ and Mical3$_{bMERB}$ complexes (PDB 5SZG)[4].

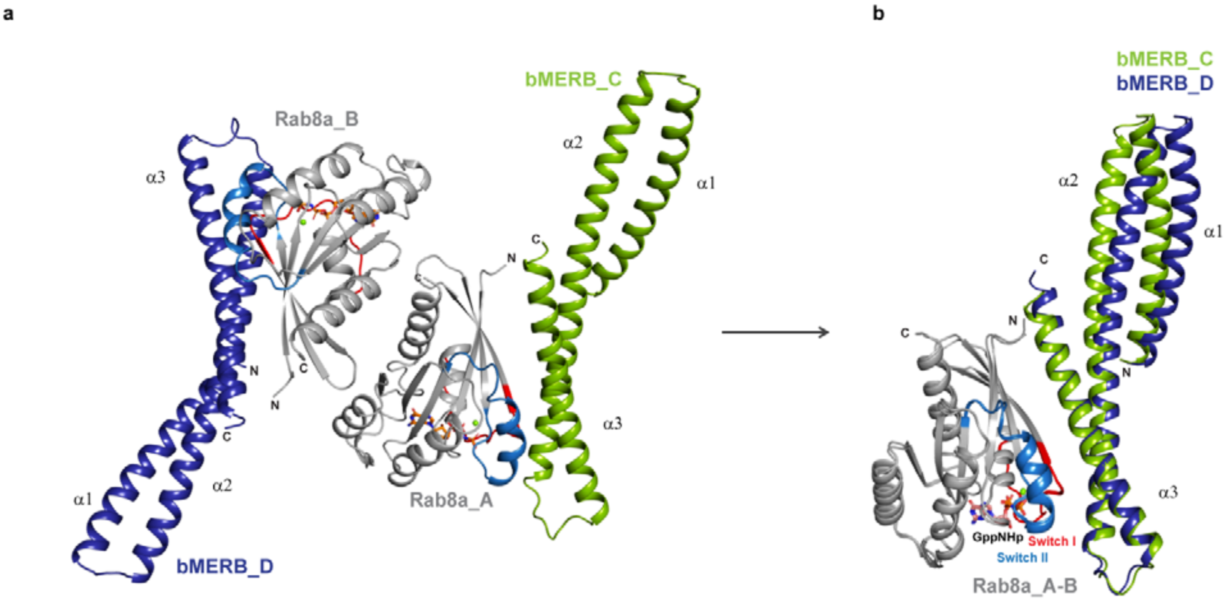

Supplementary Fig. 6: Structure of EHBP1 bMERB$_{F1120A}$:Rab8a$_{GppNHp}$ complex. (**a**) The asymmetric unit of bMERB$_{F1120A}$:Rab8a$_{GppNHp}$ complex is comprised of two complexes of each of bMERB (blue/green) and Rab8a (gray). (**b**) Overlay of both copies of bMERB$_{F1120A}$:Rab8a$_{GppNHp}$ complex in the asymmetric unit reveals that the Rab8a binding site is quite conserved; however, helix 1 and 2 adopt slightly different conformations, indicating intrinsic flexibility. Switch I and switch II are shown in red and blue, respectively. GppNHp and Mg^{2+} are shown as sticks and a green sphere, respectively.

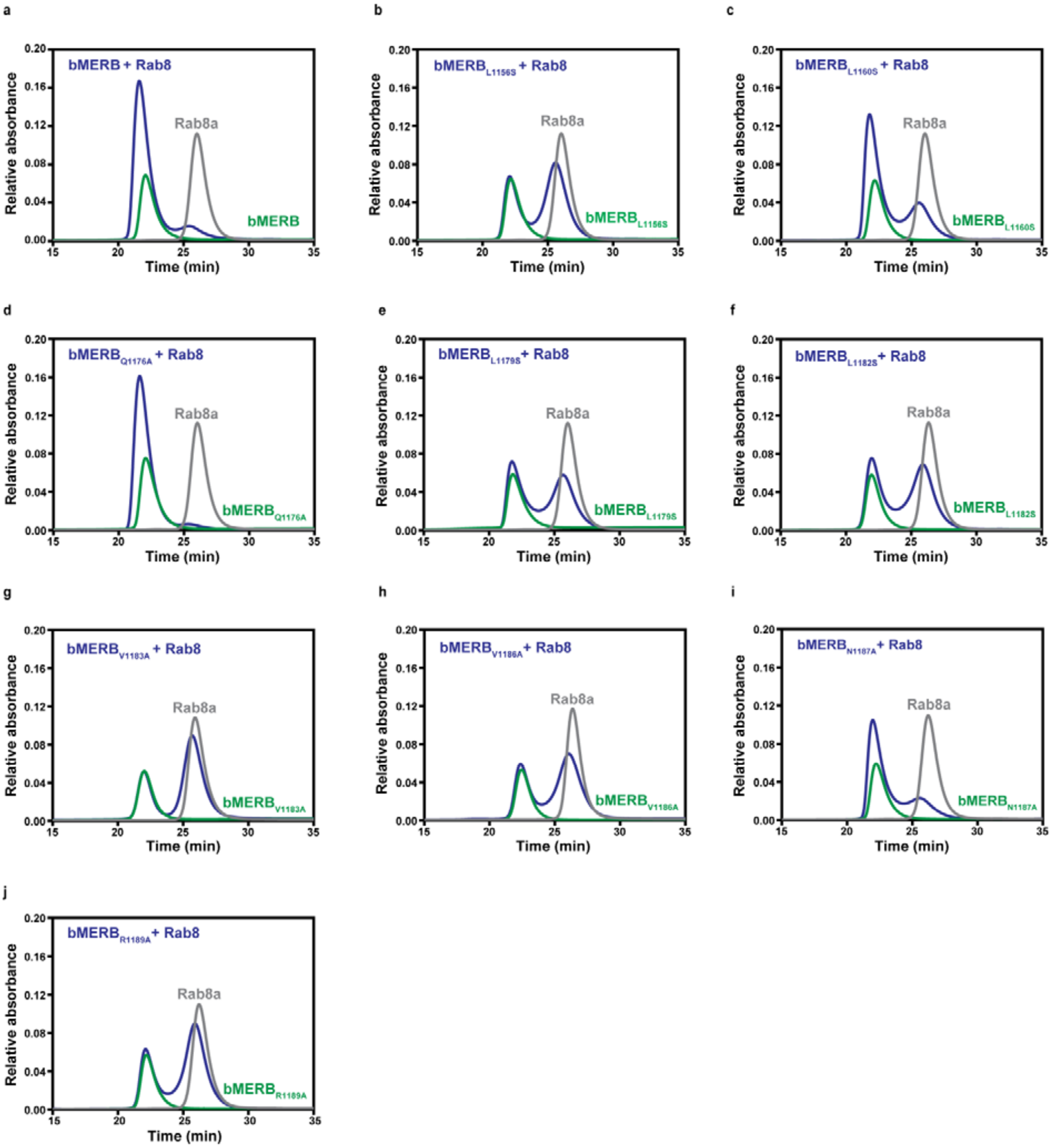

Supplementary Fig. 7: Characterization of Rab8a binding site mutants of the bMERB domain. (**a-j**) Binding of GppNHp Rab8a$_{1-176}$ (gray) with different EHBP1 bMERB mutants (green) was systematically tested onto a Superdex75 10/300 GL column. Mutants L1160S, Q1176A and N1187A form clear complexes. The bMERB constructs having low Rab8a binding affinity (L1156S, L1179S, L1182S, V1183A, V1186A and R1189A) failed to form stable complexes.

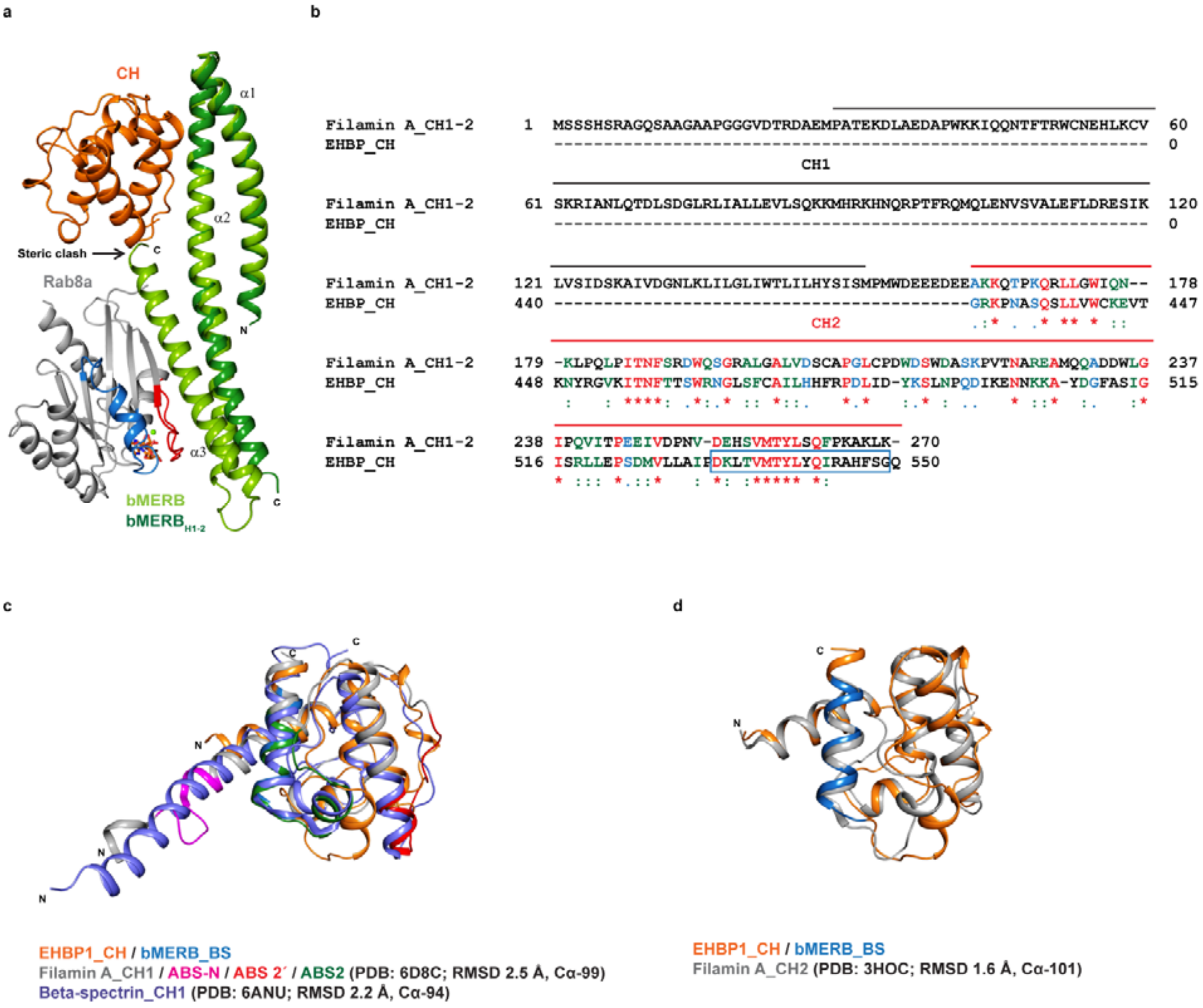

Supplementary Fig. 8: Structural basis of the CH domain release from the bMERB domain upon Rab8a binding. (**a**) Structural superposition of the EHBP1 bMERB$_{H1-2}$:CH domain and EHBP1 bMERB$_{M1116A}$:Rab8a$_{GppNHp}$ (Chain A and Chain C) complexes. (**b**) Sequence alignment of the EHBP1 CH domain with the filamin A CH1-CH2 domain using Clustal Omega. bMERB domain interacting region of the EHBP1 CH domain is shown in the light blue box. (**c**) Structural alignment of the EHBP1 CH domain (orange, bMERB binding site (bMERB_BS) in blue) with the filamin A CH1 domain (gray, PDB 6D8C)[5] and the beta-III-spectrin CH1 domain (lavender, PDB 6ANU)[6]. F-actin binding sites in the filamin A CH1 domain are shown in pink (ABS-N), red (ABS2´), and in green (ABS2). (ABS: actin binding site) (**d**) Structural superposition of the of EHBP1 CH domain (orange, bMERB binding site [bMERB_BS] in blue) with the filamin A CH2 domain (gray, PDB 3HOC)[7]. EHBP1 CH domain is similar to the filamin A CH2 domain.

Supplementary table1: Data-collection and refinement statistics (values in parentheses are for the outer shell).

	$bMERB_{H1-2}$:CH	$bMERB_{M1116A}$:Rab8a	$bMERB_{F1120A}$:Rab8a
**Data collection#**			
X-Ray Source	X10SA at SLS	X10SA at SLS	X10SA at SLS
Wavelength (Å)	1.000010	0.919532	0.919550
Resolution range (Å)	44.81–2.2 (2.279–2.2)	46.11–1.914 (1.983–1.914)	42.14 - 2.0 (2.072–2.0)
Space group	$P2_1$	C2	C2
Unit cell<br>a,b,c (Å)<br>α, β, γ (°)	54.06, 48.19, 100.30<br>90, 97.481, 90	116.36, 35.38, 165.67<br>90, 93.76, 90	117.34, 35.66, 168.56<br>90, 94.5, 90
Total reflections	173610 (17527)	591368 (32821)	316541 (30464)
Unique reflections	26306 (2604)	52600 (4963)	47864 (4700)
Multiplicity	6.5 (6.7)	11.2 (6.6)	6.6 (6.5)
Completeness (%)	99.80 (99.92)	99.46 (94.88)	98.99 (99.68)
Mean I/sigma(I)	11.83 (1.92)	19.75 (2.17)	13.62 (1.16)
Wilson B-factor	41.48	41.46	48.04
R_{merge} (%)	0.1226 (1.359)	0.06805 (0.8495)	0.06788 (1.36)
R_{meas} (%)	0.1334 (1.475)	0.07097 (0.9219)	0.07393 (1.481)
CC1/2	0.998 (0.700)	1 (0.778)	0.999 (0.5607)
CC*	0.999 (0.907)	1 (0.936)	1 (0.869)
**Refinement**			
Resolution range (Å)	44.82–2.2 (2.257–2.2)	46.11–1.914 (1.949–1.914)	42.14 - 2.0 (2.04 - 2.0)
Reflections used in refinement	26301 (2605)	52591 (4963)	47480 (4688)
Reflections used for R-free	1316 (131)	2630 (248)	2383 (217)
R_{work} (%)	0.1825 (0.2789)	0.2235 (0.3143)	0.2475 (0.4234)
R_{free} (%)	0.2213 (0.2963)	0.2670 (0.3563)	0.2908 (0.4648)
Number of non-hydrogen atoms<br>Macromolecules<br>Ligands<br>water	3320<br>3190<br>-<br>130	5111<br>4989<br>80<br>42	5181<br>4971<br>66<br>144
R.m.s deviations<br>Bond length (Å)<br>Bond angles (°)	0.015<br>1.60	0.010<br>1.24	0.010<br>1.19
Ramachandran plot<br>Favored (%)<br>Additionally allowed (%)<br>Outliers (%)	97.89<br>2.11<br>-	97.83<br>2.17<br>-	98.16<br>1.84<br>-
B-factors ($Å^2$)<br>Protein<br>Ligands<br>Water	52.79<br>52.91<br>-<br>49.82	70.54<br>71.26<br>39.25<br>44.85	72.31<br>73.23<br>36.57<br>57.02
PDB ID	6ZSH	6ZSI	6ZSJ

All data sets were collected from one single crystal on beamline X10SA of the Swiss Light Source (Paul Scherrer Institute, Villigen, Switzerland)

Supplementary table2: Expression constructs used in this study.

Construct	Plasmid	Description	Insert boundaries (restriction sites)	Purification	Purpose
EGFP-EHBP1-FL	pEGFP (C1)-hs-EHBP1	EGFP- hs-EHBP1 isform3-fl	aa 1-1160 Isoform 3 (XhoI/SmaI)		Transient expression
EGFP-EHBP1 ΔNT-C2	pEGFP (C1)-hs-EHBP1 ΔNT-C2	EGFP-hs- EHBP1 isform3 lack NT-C2 domain	aa 161-1160 Isoform 3 (XhoI/SmaI)		Transient expression
EGFP-EHBP1 ΔCaaX	pEGFP (C1) EHBP1 ΔCaaX	EGFP- EHBP1 isform3 lack CaaX	aa 1-1156 Isoform 3 (XhoI/SmaI)		Transient expression
NT-C2-EGFP	pEGFP (N1)-hs-NT-C2	Hs-NT-C2 domain-EGFP	aa 1-164 (XhoI/SmaI)		Transient expression
mCherry-Rab8a$_{Q67L}$	pmCherry (N1)-hs-Rab8a$_{Q67L}$	Rai et al., 2016[4]			Transient expression
mCherry-Rab10$_{Q68L}$	pmCherry (N1)-hs-Rab10$_{Q68L}$	Rai et al., 2016[4]			Transient expression
His-MBP-NT-C2 domain	pMAL-hs-NT-C2 domain	6xHis-MBP-TEV-hs-NT-C2domain	aa 12-164 (NdeI / XhoI)	Ni-NTA, TEV, Ni-NTA, Superdex75	Lipid overlay
His-MBP	pMAL	6His-MBP-TEV		Ni-NTA, Superdex75	Lipid overlay
Rab8a	pET19mod-hs-Rab8a$_{opti}$	Blemiling et al., 2008[8]	Full-length codon optimized (NdeI / XhoI)	Ni-NTA, TEV, Ni-NTA, Superdex75	Stopped-flow, Crystallization
Rab8a$_{1\text{-}176}$	pET19mod-hs-Rab8a$_{1\text{-}176opti}$	Rai et al., 2016[4]	aa 1-176 codon optimized (NdeI / XhoI)	Ni-NTA, TEV, Ni-NTA, Superdex75	aSEC, ITC, Crystallization
Rab8a$_{1\text{-}176_T72E}$	pET19mod-hs-Rab8a$_{1\text{-}176_T72E\ opti}$	6His-TEV-hs-Rab8a$_{1\text{-}176_T72E\ opti}$	aa 1-175 codon optimized Quick change to mutate T72 to E	Ni-NTA, TEV, Ni-NTA, Superdex75	aSEC, ITC
Rab10$_{1\text{-}175}$	pET19mod-hs-Rab10$_{1\text{-}175\ opti}$	Rai et al., 2016[4]	aa 1-175 codon optimized (NdeI / XhoI)	Ni-NTA, TEV, Ni-NTA, Superdex75	aSEC, ITC
Rab10$_{1\text{-}175_T73E}$	pET19mod-hs-Rab8a$_{1\text{-}175_T73E\ opti}$	6His-TEV-Rab10$_{1\text{-}175_T73E\ opti}$	aa 1-175 codon optimized Quick change to mutate T73 to E	Ni-NTA, TEV, Ni-NTA, Superdex 75	aSEC, ITC
GroES_EL	pGroESL	Goloubinoff et al., 1989[9]			Co-expression with Rab
EHBP1 CH	pET19mod-hs-EHBP1 CH	6xHis-TEV-hs-EHBP1$_{440\text{-}550}$ (CH domain)	aa 440-550 (NdeI / XhoI)	Ni-NTA, TEV, Ni-NTA, Superdex 75	Crystallization
EHBP1 CH	pET19mod-hs-NCys EHBP1 CH	6xHis-TEV-hs-EHBP1$_{440\text{-}550}$ (CH domain)	aa 440-550 (NdeI / XhoI)	Ni-NTA, TEV, Ni-NTA, Superdex 75	aSEC, ITC, Stopped-flow,
EHBP1 CH$_{D532A}$	pET19mod-hs-NCys EHBP1 CH$_{D532A}$	6xHis-TEV-hs-EHBP1$_{440\text{-}550_D532A}$ (CH domain)	aa 440-550 (NdeI / XhoI) Quick change to mutate D532 to A	Ni-NTA, TEV, Ni-NTA, Superdex 75	ITC
EHBP1 CH$_{L534A}$	pET19mod-hs-NCys EHBP1 CH$_{L534A}$	6xHis-TEV-hs-EHBP1$_{440\text{-}550_L534A}$ (CH domain)	aa 440-550 (NdeI / XhoI) Quick change to mutate L534 to A	Ni-NTA, TEV, Ni-NTA, Superdex 75	ITC
EHBP1 CH$_{M537A}$	pET19mod-hs-NCys EHBP1 CH$_{M537A}$	6xHis-TEV-hs-EHBP1$_{440\text{-}550_M537A}$	aa 440-550 (NdeI / XhoI)	Ni-NTA, TEV, Ni-NTA, Superdex 75	ITC

		(CH domain)	Quick change to mutate M537 to A		
EHBP1 CH$_{L534A}$	pET19mod-hs-NCys EHBP1 CH$_{T538A}$	6xHis-TEV-hs-EHBP1$_{440\text{-}550_T538A}$ (CH domain)	aa 440-550 (NdeI / XhoI) Quick change to mutate T538 to A	Ni-NTA, TEV, Ni-NTA, Superdex 75	ITC
EHBP1 CH$_{Y541A}$	pET19mod-hs-NCys EHBP1 CH$_{Y541A}$	6xHis-TEV-hs-EHBP1$_{440\text{-}550_Y541A}$ (CH domain)	aa 440-550 (NdeI / XhoI) Quick change to mutate Y541 to A	Ni-NTA, TEV, Ni-NTA, Superdex 75	ITC
EHBP1 CH$_{Q542A}$	pET19mod-hs-NCys EHBP1 CH$_{Q542A}$	6xHis-TEV-hs-EHBP1$_{440\text{-}550_Q542A}$ (CH domain)	aa 440-550 (NdeI / XhoI) Quick change to mutate Q542 to A	Ni-NTA, TEV, Ni-NTA, Superdex 75	ITC
EHBP1 bMERB	pET19mod-hs-EHBP1$_{bMERB}$	6xHis-TEV-hs-EHBP1$_{1060\text{-}1212}$ (bMERB domain)	aa 1060-1212 (NdeI / XhoI)	Ni-NTA, TEV, Ni-NTA, Superdex 75	aSEC, ITC, Stopped-flow, Crystallization
EHBP1 bMERB$_{H1\text{-}2}$	pET19mod-hs-EHBP1$_{H2(1\text{-}2)}$	6xHis-TEV-hs-EHBP1$_{1060\text{-}1162}$ (Helix 1-2 of bMERB domain)	aa 1060-1162 (NdeI / XhoI)	Ni-NTA, TEV, Ni-NTA, Superdex 75	aSEC, ITC, Crystallization
EHBP1 bMERB$_{H2\text{-}3}$	pET19mod-hs-EHBP1$_{H2(2\text{-}3)}$	6xHis-TEV-hs-EHBP1$_{1108\text{-}1212}$ (Helix 2-3 of bMERB domain)	aa 1108-1212 (NdeI / XhoI)	Ni-NTA, TEV, Ni-NTA, Superdex 75	aSEC, ITC, Crystallization
EHBP1 bMERB$_{L1099A_R110A}$	pET19mod-hs-EHBP1$_{bMERB__LR_AA}$	6xHis-TEV-hs-EHBP1$_{1060\text{-}1212_L1099A_R1100A}$ (bMERB domain)	aa 1060-1212 (NdeI / XhoI) Quick change to mutate L1099 and R1100 to A	Ni-NTA, TEV, Ni-NTA, Superdex 75	aSEC, ITC, Crystallization
EHBP1 bMERB$_{M1103A}$	pET19mod-hs-EHBP1$_{bMERB_M1103A}$	6xHis-TEV-hs-EHBP1$_{1060\text{-}1212_M1103A}$ (bMERB domain)	aa 1060-1212 (NdeI / XhoI) Quick change to mutate M1103 to A	Ni-NTA, TEV, Ni-NTA, Superdex 75	aSEC, ITC, Crystallization
EHBP1 bMERB$_{M1116A}$	pET19mod-hs-EHBP1$_{bMERB_M1116A}$	6xHis-TEV-hs-EHBP1$_{1060\text{-}1212_M1116A}$ (bMERB domain)	aa 1060-1212 (NdeI / XhoI) Quick change to mutate M1116 to A	Ni-NTA, TEV, Ni-NTA, Superdex 75	aSEC, ITC, Crystallization
EHBP1 bMERB$_{W1119A}$	pET19mod-hs-EHBP1$_{bMERB_W1119A}$	6xHis-TEV-hs-EHBP1$_{1060\text{-}1212_W1119A}$ (bMERB domain)	aa 1060-1212 (NdeI / XhoI) Quick change to mutate W1119 to A	Ni-NTA, TEV, Ni-NTA, Superdex 75	aSEC, ITC, Crystallization
EHBP1 bMERB$_{F1120A}$	pET19mod-hs-EHBP1$_{bMERB_F1120A}$	6xHis-TEV-hs-EHBP1$_{1060\text{-}1212_F1120A}$ (bMERB domain)	aa 1060-1212 (NdeI / XhoI) Quick change to mutate F1120 to A	Ni-NTA, TEV, Ni-NTA, Superdex 75	aSEC, ITC, Crystallization
EHBP1 bMERB$_{L1156S}$	pET19mod-hs-EHBP1$_{bMERB_L1156S}$	6xHis-TEV-hs-EHBP1$_{1060\text{-}1212_L1156S}$ (bMERB domain)	aa 1060-1212 (NdeI / XhoI) Quick change to mutate L1156 to S	Ni-NTA, TEV, Ni-NTA, Superdex 75	aSEC, ITC
EHBP1 bMERB$_{L1160S}$	pET19mod-hs-EHBP1$_{bMERB_L1160S}$	6xHis-TEV-hs-EHBP1$_{1060\text{-}1212_L1160S}$ (bMERB domain)	aa 1060-1212 (NdeI / XhoI) Quick change to mutate L1160 to S	Ni-NTA, TEV, Ni-NTA, Superdex 75	aSEC, ITC
EHBP1 bMERB$_{Q1176A}$	pET19mod-hs-EHBP1$_{bMERB_Q1176A}$	6xHis-TEV-hs-EHBP1$_{1060\text{-}1212_Q1176A}$ (bMERB domain)	aa 1060-1212 (NdeI / XhoI) Quick change to mutate Q1176 to A	Ni-NTA, TEV, Ni-NTA, Superdex 75	aSEC, ITC, Crystallization
EHBP1	pET19mod-hs-	6xHis-TEV-hs-	aa 1060-1212	Ni-NTA, TEV, Ni-	aSEC, ITC

bMERB$_{L1179S}$	EHBP1$_{bMERB_L1179S}$	EHBP1$_{1060-1212_L1179S}$ (bMERB domain)	(NdeI / XhoI) Quick change to mutate L1179 to S	NTA, Superdex 75	
EHBP1 bMERB$_{L1182S}$	pET19mod-hs-EHBP1$_{bMERB_L1182S}$	6xHis-TEV-hs-EHBP1$_{1060-1212_L1182S}$ (bMERB domain)	aa 1060-1212 (NdeI / XhoI) Quick change to mutate L1183 to S	Ni-NTA, TEV, Ni-NTA, Superdex 75	aSEC, ITC
EHBP1 bMERB$_{V1183A}$	pET19mod-hs-EHBP1$_{bMERB_V1183A}$	6xHis-TEV-hs-EHBP1$_{1060-1212_V1183A}$ (bMERB domain)	aa 1060-1212 (NdeI / XhoI) Quick change to mutate V1183 to A	Ni-NTA, TEV, Ni-NTA, Superdex 75	aSEC, ITC
EHBP1 bMERB$_{V1186A}$	pET19mod-hs-EHBP1$_{bMERB_V1186A}$	6xHis-TEV-hs-EHBP1$_{1060-1212_V1186A}$ (bMERB domain)	aa 1060-1212 (NdeI / XhoI) Quick change to mutate V1186 to A	Ni-NTA, TEV, Ni-NTA, Superdex 75	aSEC, ITC
EHBP1 bMERB$_{N1187A}$	pET19mod-hs-EHBP1$_{bMERB_N1187A}$	6xHis-TEV-hs-EHBP1$_{1060-1212_N1187A}$ (bMERB domain)	aa 1060-1212 (NdeI / XhoI) Quick change to mutate N1187 to A	Ni-NTA, TEV, Ni-NTA, Superdex 75	aSEC, ITC
EHBP1 bMERB$_{R1189A}$	pET19mod-hs-EHBP1$_{bMERB_R1189A}$	6xHis-TEV-hs-EHBP1$_{1060-1212_R1189A}$ (bMERB domain)	aa 1060-1212 (NdeI / XhoI) Quick change to mutate R1189 to A	Ni-NTA, TEV, Ni-NTA, Superdex 75	aSEC, ITC
Mical1 bMERB	pET19mod-hs-Mical1	Rai et al., 2016[4]	aa 918-1067 (NdeI / XhoI)	Ni-NTA, Precission, Ni-NTA, Superdex 75	aSEC, ITC
Mical1 CH	pET19mod-hs-MicalL1 CH	6xHis-TEV-hs-Mical1$_{506-614}$ (CH domain)	aa 506-614 (NdeI / XhoI)	Ni-NTA, TEV, Ni-NTA, Superdex 75	aSEC, ITC
Mical1 LIM	pET19mod-hs-Mical1 LIM	6xHis-TEV-hs-Mical1$_{687-757}$ (LIM domain)	aa 687-757 (NdeI / XhoI)	Ni-NTA, TEV, Ni-NTA, Superdex 75	aSEC, ITC
Mical1 CH-LIM	pET19mod-hs-Mical1 CH-LIM	6xHis-TEV-hs-Mical1$_{506-757}$ (CH-LIM domain)	aa 506-757 (NdeI / XhoI)	Ni-NTA, TEV, Ni-NTA, Superdex 75	aSEC, ITC
Mical3 bMERB	pOPINF-hs-Mical3	Rai et al., 2016	aa 1841-1990 Infusion cloning	Ni-NTA, Precission, Ni-NTA, Superdex 75	aSEC, ITC
Mical3 CH	pET19mod-hs-MicalL3 CH	6xHis-TEV-hs-Mical3$_{516-626}$ (CH domain)	aa 516-626 (NdeI / XhoI)	Ni-NTA, TEV, Ni-NTA, Superdex 75	aSEC, ITC
Mical-L1 bMERB	pET19mod-hs-Mical-L1$_{671-830}$	6xHis-TEV-hs-Mical-L1$_{671-830}$ (bMERB domain)	aa 671-830 (NdeI / XhoI)	Ni-NTA, TEV, Ni-NTA, Superdex 75	aSEC, ITC
Mical-L1 CH	pET19mod-hs-MicalL1-CH	6xHis-TEV-hs-Mical-L1$_{1-109}$ (CH domain)	aa 1-109 (NdeI / XhoI)	Ni-NTA, TEV, Ni-NTA, Superdex 75	aSEC, ITC

hs: homo sapiens, MBP: maltose binding protein

Supplementary table3: Primers used in this study.

Primer name	Primer sequence (5´ to 3´)
EHBP1_Fl_Isoform3 FP	GATCCTCGAGCTATGGCTTCAGTTTGGAAGAGACTGCAGCG
EHBP1_Fl_Isoform3 RP	GATCCCCGGGCTACTGAAGAACACATTTCTCCTCTTTCTTGGC
EHBP1_fl_Isoform3_ΔNT-C2 FP	GATCCTCGAGCTATGGAAGGAAAAGCCACAGATGAAGACA
EHBP1_fl_Isoform3_ΔCAAX RP	GATCCCCGGGCTATTTCTCCTCTTTCTTGGCCATCTTGCCTTTG
EHBP1_NT-C2 RP	GATCCCCGGGCGGCTTTTCCTTCCCTCAGAAAAATGCAAGA
EHBP1_bMERB FP	AAAAAACATATGGATGAAGTGCTTAATAAAGGGTTCAAAGAC
EHBP1_bMERB RP	AAAAAACTCGAGTTATCGCTCCAAATGCTCATCTTCTTCTTCG
EHBP1_bMERB_H12 RP	AAAAAACTCGAGTTAAATGGCTAGCATTGCCCTCAATTCCCG
EHBP1_bMERB_H23 FP	GATCCATATGAACACAGAAGAAGAAGAAGCTATGATGCAG
EHBP1_CH FP	GATCCATATGGGGCGAAAGCCAAATGCTAGTCAGTCTTTGCTTGTA
EHBP1_CH RP	GATCCTCGAGTTATTGGCCACTGAAATGTGCCCTTATTTGATAGAG
Mical1 _CH FP	GATCCATATGGGGTCGGCAGGCACCCAGGAGGAGCTGCTA
Mical1_CH_RP	GATCCTCGAGTTACATGCTCTTGAAGGCACTGTGGAAGTGGCT
Mical1_LIM_FP	GATCCATATGCAACACCAGGAGGCCGGTGCTGGGGACCTGTGTGCACTT
Mical1_LIM_RP	GATCCTCGAGTTATTTGTGGTCTGTCTGGGGCAGGTGCTGGAGGCAGTAGAA
Mical3_CH_FP	GATCCATATGGAGTCTGTAGCTCGTTCAAGCAAACTGCTG
Mical3_CH_RP	GATCCTCGAGTCAGGAGTCCTTAAACATCTCGTAGAACTGAGT
Mical-L1_bMERB FP	AAAAACATATG CGCAAGGTCCAGGCTGACCAGTACATCCC
Mical-L1_bMERB RP	AAAAACTCGAGTCA TTCAGCCTCCCTCTGGAACTCTTTCTTCTTGATCATGGC
Mical-L1_CH FP	GATCCATATGGCTGGGCCGCGGGGCGCGCTGCTGGCCTGGTGCC
Mical-L1_CH RP	GATCCTCGAGTCAACTGCAGAAGTGGTTGTAATACTGGGACAC
Rab8a T72E FP	GGTCAAGAACGCTTCCGTGAGATTACCACCGCCTATTAT
Rab8a T72E RP	ATAATAGGCGGTGGTAATCTCACGGAAGCGTTCTTGACC
Rab10 T73E FP	GGGCAAGAACGCTTTCACGAGATTACTACCAGCTATTAT
Rab10 T73E RP	ATAATAGCTGGTAGTAATCTCGTGAAAGCGTTCTTGCCC
EHBP1_CH_D532A FP	ATGGTATTATTAGCAATTCCTGCTAAACTGACTGTTATGACTTAT
EHBP1_CH_D532A RP	ATAAGTCATAACAGTCAGTTTAGCAGGAATTGCTAATAATACCAT
EHBP1_CH_L534A FP	TTATTAGCAATTCCTGATAAAGCGACTGTTATGACTTATCTCTAT
EHBP1_CH_L534A RP	ATAGAGATAAGTCATAACAGTCGCTTTATCAGGAATTGCTAATAA
EHBP1_CH_M537A FP	ATTCCTGATAAACTGACTGTTGCGACTTATCTCTATCAAATAAGG
EHBP1_CH_M537A RP	CCTTATTTGATAGAGATAAGTCGCAACAGTCAGTTTATCAGGAAT
EHBP1_CH_T538A FP	CCTGATAAACTGACTGTTATGGCTTATCTCTATCAAATAAGGGCA
EHBP1_CH_T538A RP	TGCCCTTATTTGATAGAGATAAGCCATAACAGTCAGTTTATCAGG
EHBP1_CH_Y541A FP	CTGACTGTTATGACTTATCTCGCTCAAATAAGGGCACATTTCAGT
EHBP1_CH_Y541A RP	ACTGAAATGTGCCCTTATTTGAGCGAGATAAGTCATAACAGTCAG
EHBP1_CH_Q542A FP	ACTGTTATGACTTATCTCTATGCAATAAGGGCACATTTCAGTGGC
EHBP1_CH_Q542A FP	GCCACTGAAATGTGCCCTTATTGCATAGAGATAAGTCATAACAGT
EHBP1_bMERB_L1099A_R1100A FP	GCGCTGGTGGAGAAGCGCGCTGCCTATCTCATGGACACAGGA
EHBP1_bMERB_L1099A_R1100A RP	TCCTGTGTCCATGAGATAGGCAGCGCGCTTCTCCACCAGCGC
EHBP1_bMERB_M1103A FP	GAGAAGCGCCTTCGCTATCTCGCGGACACAGGAAGGAACACAGAA
EHBP1_bMERB_M1103A FP	TTCTGTGTTCCTTCCTGTGTCCGCGAGATAGCGAAGGCGCTTCTC
EHBP1_bMERB_M1116A FP	ACAGAAGAAGAAGAAGCTATGGCGCAGGAATGGTTTATGTTAGTT
EHBP1_bMERB_M1116A RP	AACTAACATAAACCATTCCTGCGCCATAGCTTCTTCTTCTTCTGT
EHBP1_bMERB_W1119A FP	AAGCTATGATGCAGGAAGCGTTTATGTTAGTTAATAAG
EHBP1_bMERB_W1119A RP	CTTATTAACTAACATAAACGCTTCCTGCATCATAGCTT
EHBP1_bMERB_F1120A FP	GCTATGATGCAGGAATGGGCTATGTTAGTTAATAAGAAA
EHBP1_bMERB_F1120A RP	TTTCTTATTAACTAACATAGCCCATTCCTGCATCATAGC
EHBP1_bMERB_L1156S FP	GAGCTGCTGAACCGGGAATCGAGGGCAATGCTAGCCATT
EHBP1_bMERB_L1156S RP	AATGGCTAGCATTGCCCTCGATTCCCGGTTCAGCAGCTC
EHBP1_bMERB_L1160S FP	CGGGAATTGAGGGCAATGTCAGCCATTGAAGACTGGCAG
EHBP1_bMERB_L1160S RP	CTGCCAGTCTTCAATGGCTGACATTGCCCTCAATTCCCG
EHBP1_bMERB_Q1176A FP	GCCCAGAAGCGACGCGAAGCGCTTCTGCTAGATGAGCTG
EHBP1_bMERB_Q1176A RP	CAGCTCATCTAGCAGAAGCGCTTCGCGTCGCTTCTGGGC
EHBP1_bMERB_L1179S FP	CGACGCGAACAGCTTCTGTCAGATGAGCTGGTGGCCCTG

EHBP1_bMERB_L1179S RP	CAGGGCCACCAGCTCATCTGACAGAAGCTGTTCGCGTCG
EHBP1_bMERB_L1182S FP	CAGCTTCTGCTAGATGAGTCGGTGGCCCTGGTGAACAAG
EHBP1_bMERB_L1182S RP	CTTGTTCACCAGGGCCACCGACTCATCTAGCAGAAGCTG
EHBP1_bMERB_V1183A FP	CTTCTGCTAGATGAGCTGGCGGCCCTGGTGAACAAGCGC
EHBP1_bMERB_V1183A RP	GCGCTTGTTCACCAGGGCCGCCAGCTCATCTAGCAGAAG
EHBP1_bMERB_V1186A FP	GATGAGCTGGTGGCCCTGGCGAACAAGCGCGATGCGCTC
EHBP1_bMERB_V1186A RP	GAGCGCATCGCGCTTGTTCGCCAGGGCCACCAGCTCATC
EHBP1_bMERB_N1187A FP	GAGCTGGTGGCCCTGGTGGCCAAGCGCGATGCGCTCGTC
EHBP1_bMERB_N1187A RP	GACGAGCGCATCGCGCTTGGCCACCAGGGCCACCAGCTC
EHBP1_bMERB_R1189A FP	GCCCTGGTGAACAAGGCCGATGCGCTCGTCAGGGAC
EHBP1_bMERB_R1189A RP	GTCCCTGACGAGCGCATCGGCCTTGTTCACCAGGGC

References

1. Tomizawa, T., Kigawa, T., Koshiba, S., Inoue, M. & Yokoyama, S. (RIKEN Structural Genomics/Proteomics Initiative (RSGI), 2006).
2. Lee, S.H., Weins, A., Hayes, D.B., Pollak, M.R. & Dominguez, R. Crystal structure of the actin-binding domain of alpha-actinin-4 Lys255Glu mutant implicated in focal segmental glomerulosclerosis. *J Mol Biol* **376**, 317-324 (2008).
3. Djinovic Carugo, K., Banuelos, S. & Saraste, M. Crystal structure of a calponin homology domain. *Nat Struct Biol* **4**, 175-179 (1997).
4. Rai, A. *et al.* bMERB domains are bivalent Rab8 family effectors evolved by gene duplication. *Elife* **5** (2016).
5. Iwamoto, D.V. *et al.* Structural basis of the filamin A actin-binding domain interaction with F-actin. *Nat Struct Mol Biol* **25**, 918-927 (2018).
6. Avery, A.W. *et al.* Structural basis for high-affinity actin binding revealed by a beta-III-spectrin SCA5 missense mutation. *Nat Commun* **8**, 1350 (2017).
7. Clark, A.R., Sawyer, G.M., Robertson, S.P. & Sutherland-Smith, A.J. Skeletal dysplasias due to filamin A mutations result from a gain-of-function mechanism distinct from allelic neurological disorders. *Hum Mol Genet* **18**, 4791-4800 (2009).
8. Bleimling, N., Alexandrov, K., Goody, R. & Itzen, A. Chaperone-assisted production of active human Rab8A GTPase in Escherichia coli. *Protein Expr Purif* **65**, 190-195 (2009).
9. Goloubinoff, P., Gatenby, A.A. & Lorimer, G.H. GroE heat-shock proteins promote assembly of foreign prokaryotic ribulose bisphosphate carboxylase oligomers in Escherichia coli. *Nature* **337**, 44-47 (1989).
