# Supplementary material for: The mechanism of activation of the actin binding protein EHBP1 by Rab8 family members
Source: Nat Commun. 2020 Aug 21;11:4187. doi: 10.1038/s41467-020-17792-3 (PMC7442826; doi:10.1038/s41467-020-17792-3)
Supplement: Supplementary file 3 — Reporting Summary [file 41467_2020_17792_MOESM3_ESM.pdf]

nature research

Corresponding author(s): Amrita Rai and Roger Goody

Last updated by author(s): Jul 17, 2020

Reporting Summary

Nature Research wishes to improve the reproducibility of the work that we publish. This form provides structure for consistency and transparency in reporting. For further information on Nature Research policies, see our Editorial Policies and the Editorial Policy Checklist.

Statistics

For all statistical analyses, confirm that the following items are present in the figure legend, table legend, main text, or Methods section.

n/a	Confirmed	
☐	☒	The exact sample size (*n*) for each experimental group/condition, given as a discrete number and unit of measurement
☐	☒	A statement on whether measurements were taken from distinct samples or whether the same sample was measured repeatedly
☒	☐	The statistical test(s) used AND whether they are one- or two-sided<br>*Only common tests should be described solely by name; describe more complex techniques in the Methods section.*
☒	☐	A description of all covariates tested
☒	☐	A description of any assumptions or corrections, such as tests of normality and adjustment for multiple comparisons
☐	☒	A full description of the statistical parameters including central tendency (e.g. means) or other basic estimates (e.g. regression coefficient) AND variation (e.g. standard deviation) or associated estimates of uncertainty (e.g. confidence intervals)
☒	☐	For null hypothesis testing, the test statistic (e.g. *F*, *t*, *r*) with confidence intervals, effect sizes, degrees of freedom and *P* value noted<br>*Give P values as exact values whenever suitable.*
☒	☐	For Bayesian analysis, information on the choice of priors and Markov chain Monte Carlo settings
☒	☐	For hierarchical and complex designs, identification of the appropriate level for tests and full reporting of outcomes
☒	☐	Estimates of effect sizes (e.g. Cohen's *d*, Pearson's *r*), indicating how they were calculated

Our web collection on statistics for biologists contains articles on many of the points above.

Software and code

Policy information about availability of computer code

Data collection	X-ray diffraction data were collected on synchrotron beamline X10SA at the Swiss Light Source (Paul Scherrer Institute, Villigen, Switzerland).
Data analysis	X-ray datasets were processed and reduced with XDS package. Initial phasing was done by molecular replacement with PHASER and the atomic models were built with COOT and refined in PHENIX and REFMAC. Structural figures were prepared using PyMOL (DeLano Scientific; http://www.pymol.org). The quantitative analysis of SDS-PAGE was measured and calculated using Bio-Rad image analysis software. Graphpad Prism 7 is used to calculate the value of mean and standard deviation presented in figure 4b. Isothermal titration calorimetry data analysis was done using the MicroCal software (Origin 7). All other data were analyzed with Origin9.Additional software including Schrodinger suite and imagej 1.51s were used.

For manuscripts utilizing custom algorithms or software that are central to the research but not yet described in published literature, software must be made available to editors and reviewers. We strongly encourage code deposition in a community repository (e.g. GitHub). See the Nature Research guidelines for submitting code & software for further information.

Data

Policy information about availability of data

All manuscripts must include a data availability statement. This statement should provide the following information, where applicable:

- Accession codes, unique identifiers, or web links for publicly available datasets
- A list of figures that have associated raw data
- A description of any restrictions on data availability

The plasmids created in this study are available from the corresponding author upon reasonable request. Protein coordinates and structure factors have been submitted to the Protein Data Bank under accession codes 6ZSH (bMERB_H1-2:CH), 6ZSI (bMERB_M1116A:Rab8a), and 6ZSJ (bMERB_F1120A:Rab8a).

Field-specific reporting

Please select the one below that is the best fit for your research. If you are not sure, read the appropriate sections before making your selection.

☒ Life sciences ☐ Behavioural & social sciences ☐ Ecological, evolutionary & environmental sciences

For a reference copy of the document with all sections, see nature.com/documents/nr-reporting-summary-flat.pdf

Life sciences study design

All studies must disclose on these points even when the disclosure is negative.

Sample size	Sample size was chosen based on the standard practices in the field. No statistical methods were used to predetermine the sample size.
Data exclusions	No data was exclusion
Replication	All biochemical and cellular assays are repeated at least three times with similar results, unless otherwise stated. The number of replicate for each experiment is shown in the figure legends or method. Actin co-sedimentation experiments were performed twice independently.
Randomization	All samples were tested under same experimental conditions therefore no randomization is done in the study.
Blinding	No aspects of the study were blinded.

Reporting for specific materials, systems and methods

We require information from authors about some types of materials, experimental systems and methods used in many studies. Here, indicate whether each material, system or method listed is relevant to your study. If you are not sure if a list item applies to your research, read the appropriate section before selecting a response.

Materials & experimental systems

n/a	Involved in the study	
☐	☒	Antibodies
☐	☒	Eukaryotic cell lines
☒	☐	Palaeontology and archaeology
☒	☐	Animals and other organisms
☒	☐	Human research participants
☒	☐	Clinical data
☒	☐	Dual use research of concern

Methods

n/a	Involved in the study	
☒	☐	ChIP-seq
☒	☐	Flow cytometry
☒	☐	MRI-based neuroimaging

Antibodies

Antibodies used	anti-His antibody 1119224160001 (mouse IgG1, Sigma/Merck) and goat anti-mouse IgG HRP 10004302 (Cayman chemicals).
Validation	https://www.sigmaaldrich.com/catalog/product/sigma/sab1305538?lang=de&region=DE<br>https://www.caymanchem.com/product/10004302/goat-anti-mouse-igg-hrp

Eukaryotic cell lines

Policy information about cell lines

Cell line source(s)	COS-7 (ATCC® CRL-1651™)
Authentication	Cells were not authenticated.
Mycoplasma contamination	Not tested for mycoplasma contamination.
Commonly misidentified lines (See ICLAC register)	No commonly misidentified cell lines were used.
