# Supplementary material for: The mechanism of activation of the actin binding protein EHBP1 by Rab8 family members
Source: Nat Commun. 2020 Aug 21;11:4187. doi: 10.1038/s41467-020-17792-3 (PMC7442826; doi:10.1038/s41467-020-17792-3)
Supplement: Supplementary file 2 — Peer Review File [file 41467_2020_17792_MOESM2_ESM.docx]

Peer Review Information

**Journal:** Nature Communications

**Manuscript title:** The mechanism of activation of the actin binding protein EHBP1 by Rab8 family members

**Corresponding author name(s):** Prof Roger Goody

**Editorial notes:**

**none**

**Reviewer comments & decisions:**

**Reviewer comments, first version:**

Reviewer #1 (Remarks to the Author):

EHBP1 is an adapter protein that was originally shown by Michael Czech's group to couple endocytosis to actin binding and has recently gained significant interest based on roles in cancer, lipophagy, and stress responses. Nevertheless, key mechanistic details regarding the regulation and functions of EHBP1 in macromolecular assemblies have remained elusive. The work is considered of significant interest to a broad audience of cell biologists to individuals working on cancer therapeutics.

The manuscript by Rai et al. provides comprehensive biochemical, molecular, and structural analyses of the EHBP1 protein that shed light on the mechanisms of membrane binding, autoinhibition and activation by the Rab8 GTPase. While the early work by the Czech group defined an N-terminal membrane binding domain in EHBP1, the present study demonstrates, through mutational analyses, that a C-terminal farnesylated CAAX box is indispensable for membrane binding. This raised the question as to if and how this dually membrane tethered protein might be autoregulated in its actin and GTPase binding activities. Through a series of rigorous in vitro biochemical assays using purified wild-type and mutant CH and bMERB domains the authors unequivocally demonstrate that the EHBP1 bMERB domain forms an autoinhibited structure by binding the actin-binding CH domain. The autoinhibition is released by active, GTP-bound Rab8, a mechanism that is shown to be unique to the EHBP1 protein relative to other family members. Crystallographic analyses of the CH-bMERB and the bMERB:Rab8a complexes further affirm a mutually exclusive binding interface. The work is technically sound, comprehensive in its scope and of broad interest. Therefore, I enthusiastically recommend.

One minor comments pertains to Fig. 7b-c. The figure legend mentions Rab8a binding residues labeled in blue—this is not visible and it is hard to figure out the transformation of the view between 7a and 7b.


Reviewer #2 (Remarks to the Author):

The manuscript by Rai et al. reports a number of interesting biochemical and structural observations related to membrane recruitment of the actin binding protein EHBP1 and the molecular mechanisms for regulation of its actin binding activity through autoinhibition of the CH domain by the bMERB domain, which can be relieved by Rab8 binding. The biochemical observations clearly delineate the relevant intra/inter-molecular interactions including mutually exclusive binding of the bMERB domain to Rab8 vs. the CH domain. In addition to details of the molecular interfaces, the structural observations based on new crystal structures of the bMERB domain bound to the CH domain or Rab8 are supported by structure-based mutational analyses and clearly explain why the bMERB-CH interaction is autoinhibitory and how Rab8 binding relieves autoinhibition.

Overall, the experiments and analyses are very high quality and provide important new insights into the mechanisms of membrane recruitment and regulation of actin binding activity that represent a substantial advance. The manuscript is generally well written and I have only a few relatively minor comments.

The text states that three isoforms of EHBP1 have been reported but no reference is given. Are the authors referring to isoforms in a data base? If so, which data base(s)? If not, the reference(s) for the reported isoform should be cited.

Clarify the rationale for using isoform 1 for biochemical studies and isoform 3 for cell localization.

The results of the lipid overlay experiments (PIP strips) are potentially interesting and suggestive of stereoselective binding to PI3P/PI5P. The observations are, however, preliminary since lipid overlay assays do not always correlate with PIP-dependent membrane partitioning and PIP binding/selectivity was not further analyzed in a membrane context (e.g. liposomes with low mole fractions of PIPs). This caveat and the need for additional experiments should be discussed when presenting the experiments.

ITC provides information about binding stoichiometry (N), enthapy and entropy in addition to affinity. The authors may want to add a brief comment about stoichiometry and possibly enthalpic vs. entropic contributions if they think it is relevant.

Line 182-132, the phrase "Sequence alignment with our previous structural data" is confusing and needs clarification. Do the authors mean a structure-based alignment, a homology model, or something else?

David Lambright

**Author rebuttal, first version:**

Reviewer #1 (Remarks to the Author):

EHBP1 is an adapter protein that was originally shown by Michael Czech's group to couple endocytosis to actin binding and has recently gained significant interest based on roles in cancer, lipophagy, and stress responses. Nevertheless, key mechanistic details regarding the regulation and functions of EHBP1 in macromolecular assemblies have remained elusive. The work is considered of significant interest to a broad audience of cell biologists to individuals working on cancer therapeutics.

The manuscript by Rai et al. provides comprehensive biochemical, molecular, and structural analyses of the EHBP1 protein that shed light on the mechanisms of membrane binding, autoinhibition and activation by the Rab8 GTPase. While the early work by the Czech group defined an N-terminal membrane binding domain in EHBP1, the present study demonstrates, through mutational analyses, that a C-terminal farnesylated CAAX box is indispensable for membrane binding. This raised the question as to if and how this dually membrane tethered protein might be autoregulated in its actin and GTPase binding activities. Through a series of rigorous in vitro biochemical assays using purified wild-type and mutant CH and bMERB domains the authors unequivocally demonstrate that the EHBP1 bMERB domain forms an autoinhibited structure by binding the actin-binding CH domain. The autoinhibition is released by active, GTP-bound Rab8, a mechanism that is shown to be unique to the EHBP1 protein relative to other family members. Crystallographic analyses of the CH-bMERB and the bMERB:Rab8a complexes further affirm a mutually exclusive binding interface. The work is technically sound, comprehensive in its scope and of broad interest. Therefore, I enthusiastically recommend.

We thank the reviewer for the positive comments.

One minor comments pertain to Fig. 7b-c. The figure legend mentions Rab8a binding residues labeled in blue—this is not visible and it is hard to figure out the transformation of the view between 7a and 7b.

As per the reviewer's suggestion, we modified the figure and marked the CH and Rab8 binding sites and also labeled Rab8a residues in red. To make a clear transformation between figure 7a and 7b a new figure legend has been introduced.

**7b-c** Schematic presentation of the CH (green, N-terminal) and Rab (blue, C-terminal) binding site of EHBP1 bMERB domain. Structural and sequence alignment of the N and C-terminal halves of the EHBP1 bMERB domain, showing strong conservation between both CH and Rab8a binding sites. CH and Rab8 interacting residues are shown in red. * denotes critical residues for the interaction.

Reviewer #2 (Remarks to the Author):

The manuscript by Rai et al. reports a number of interesting biochemical and structural observations related to membrane recruitment of the actin binding protein EHBP1 and the molecular mechanisms for regulation of its actin binding activity through autoinhibition of the CH domain by the bMERB domain, which can be relieved by Rab8 binding. The biochemical observations clearly delineate the relevant intra/inter-molecular interactions including mutually exclusive binding of the bMERB domain to Rab8 vs. the CH domain. In addition to details of the molecular interfaces, the structural observations based on new crystal structures of the bMERB domain bound to the CH domain or Rab8 are supported by structure-based mutational analyses and clearly explain why the bMERB-CH interaction is autoinhibitory and how Rab8 binding relieves autoinhibition.

Overall, the experiments and analyses are very high quality and provide important new insights into the mechanisms of membrane recruitment and regulation of actin binding activity that represent a substantial advance. The manuscript is generally well written and I have only a few relatively minor comments.

We thank the reviewer for the positive comments.

The text states that three isoforms of EHBP1 have been reported but no reference is given. Are the authors referring to isoforms in a data base? If so, which data base(s)? If not, the reference(s) for the reported isoform should be cited.

We apologize to the reviewer for the confusion. In the revised manuscript, we have mentioned that the UniProt database has reported the three EHBP1 isoforms.

Clarify the rationale for using isoform 1 for biochemical studies and isoform 3 for cell localization.

UniProt database reports isoform 1 as the canonical isoform; however, we could not clone isoform 1, only isoform 3. To keep uniformity with our previously published work on EHBP1 bMERB domain^1^, for biochemical experiments, we kept the domain boundaries from isoform 1.

The results of the lipid overlay experiments (PIP strips) are potentially interesting and suggestive of stereoselective binding to PI3P/PI5P. The observations are, however, preliminary since lipid overlay assays do not always correlate with PIP-dependent membrane partitioning and PIP binding/selectivity was not further analyzed in a membrane context (e.g. liposomes with low mole fractions of PIPs). This caveat and the need for additional experiments should be discussed when presenting the experiments.

We thank the reviewer for pointing this out. We have rephrased our statement, according to the reviewer's suggestion and explained that the reported lipid overlay experiment is a preliminary observation hinting that the EHBP1 NT-C2 associates with PI3P/PI5P and PS; however, further validation is needed by lipid co-sedimentation and other biochemical experiments.

ITC provides information about binding stoichiometry (N), enthalpy and entropy in addition to affinity. The authors may want to add a brief comment about stoichiometry and possibly enthalpic vs. entropic contributions if they think it is relevant.

We thank the reviewers for raising this point. We did mention the stoichiometry; both the CH and Rab8a bind to bMERB domain with a stoichiometry of 1:1 and both the binding processes are enthalpy-driven. In the revised version, we have modified the text.

Using ITC measurements, binding was observed between full-length bMERB domain and CH domains and similar to Rab8a, CH domain binding to bMERB domain is an enthalpy driven process with a K_D_ value of 1.2 µM and stoichiometry of 1:1.

Line 182-132, the phrase "Sequence alignment with our previous structural data" is confusing and needs clarification. Do the authors mean a structure-based alignment, a homology model, or something else?

We apologize for the confusion and thankful to the reviewer for pointing this out. The text has been modified.

A structure-based sequence alignment shows that the switch II threonine of Rab8a/10 does not directly interact with Mical1_bMERB_ and Mical cL_bMERB_.

David Lambright

In addition to the changes suggested by the reviewers, we have some minor changes:

1: Within the constraints of 5000 words, we have reduced the text size, mainly the introduction.

2: We have removed the bold fonts and minor ticks from the figures and some minor modification in figure 6 and supplementary figure 5.

3: Centrifugation speed is now reported in RCF (xg).

4: Mass spectrometry data of Cy3 labelling of EHBP1CH domain are added to supplementary Fig. 2k.

**Reference**

1.	Rai, A. et al. bMERB domains are bivalent Rab8 family effectors evolved by gene duplication. *Elife* **5**(2016).

**Reviewer comments, second version:**

**Author rebuttal, second version:**

**Reviewer comments, third version:**

**Author rebuttal, third version:**
